# Receptor Allosterism Governs the Coding of Configurational Walnut Aroma

**DOI:** 10.1021/acs.jafc.6c10400

**Published:** 2026-10-29

**Authors:** Ronja Hopf, Franziska Haag, Stephanie Frank, Sandra Hoffmann, Martin Steinhaus, Dietmar Krautwurst

**Affiliations:** † TUM School of Life Sciences, 9184 Technical University of Munich, 85354 Freising, Germany; ‡ Leibniz Institute for Food Systems Biology at the Technical University of Munich, 85354 Freising, Germany

**Keywords:** GPCR, aldehyde receptor, odor coding, (2*E*,4*E*,6*Z*)-nona-2,4,6-trienal, 3-hydroxy-4,5-dimethylfuran-2(5*H*)-one (sotolon)

## Abstract

Walnut aroma is a configurational odor identity, primarily attributed to a walnut-typical 1:1 concentration ratio of the key food odorants (KFO) (2*E*,4*E*,6*Z*)-nona-2,4,6-trienal and 3-hydroxy-4,5-dimethylfuran-2­(5*H*)-one (sotolon), which by themselves impart oatmeal- and fenugreek-like odor notes, respectively. OR8D1 has been identified as a sotolon-responsive receptor, recently. A receptor for (2*E*,4*E*,6*Z*)-nona-2,4,6-trienal was unknown. We screened a collection of 766 odorant receptor variants with (2*E*,4*E*,6*Z*)-nona-2,4,6-trienal, and OR2J2 solely emerged as a responder. Testing OR2J2 with 190 KFOs and series of aldehydes revealed its KFO-biased, aldehyde-centered, and EC_50_-ranked spectrum of 47 agonists, with (2*E*,4*E*,6*Z*)-nona-2,4,6-trienal as the most potent and efficient agonist. Concentration–response relationships of (2*E*,4*E*,6*Z*)-nona-2,4,6-trienal, sotolon, and their 1:1 mixtures on OR2J2 and OR8D1 demonstrated (2*E*,4*E*,6*Z*)-nona-2,4,6-trienal as negative allosteric modulator significantly attenuating the efficacy of sotolon at OR8D1. In summary, our findings show that configurational walnut aroma is orthosterically and allosterically encoded, demonstrating yet another way odorants interact at receptors to produce novel response patterns.

## Graphical abstract

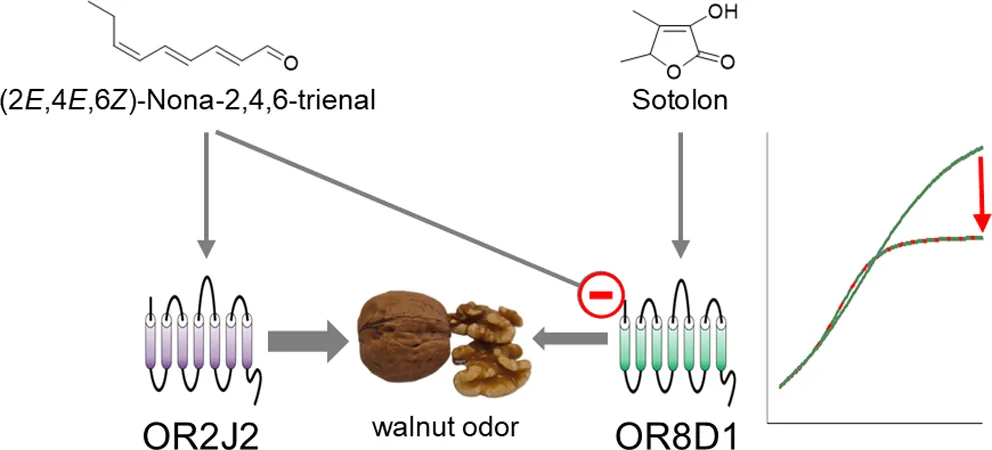

## Introduction

The global domestic consumption of walnuts amounted to over 2.6 million metric tons in 2024.^1^ Raw or roasted walnut kernels are consumed as snacks and are a common ingredient in bakery products and sweets. Fresh walnut kernels are appreciated for their characteristic aroma, which is distinctly different from, e.g., almonds or hazelnuts. Recently, a study by Stübner and Steinhaus (2023)^2^ aimed to identify and understand the characteristic aroma of walnuts. They showed that the aroma of unprocessed walnuts can be attributed to the compounds (2*E*,4*E*,6*Z*)-nona-2,4,6-trienal and 3-hydroxy-4,5-dimethyl-furan-2­(5*H*)-one (sotolon).^2^ Interestingly, both compounds had not been detected in walnuts before, although molecular sensory science approaches had been applied in previous studies. Both compounds have their own, characteristic and nonoverlapping fenugreek- and oatmeal-like odor qualities, for sotolon and (2*E*,4*E*,6*Z*)-nona-2,4,6-trienal, respectively. Both odorants as a binary mixture at walnut-typical 1:1 concentration ratio, however, elicit walnut aroma as a new, dominant odor identity,^2^ which, therefore, is truly configurational.^3^

A configurational aroma refers to an olfactory perception when a mixture of distinct odorants acts together. Instead of smelling the odor of individual components separately, the brain perceives a new smell identity.^3^ The perception of configurational odor identities has been attributed to central neural processing.^4–6^ Peripheral pharmacodynamic processes, however, such as orthosteric antagonism, partial and inverse agonism, and enhancement have been described to shape odorant coding already at the receptor level.^7–9^ Beyond, allosteric modulation, via an interaction site that differs from the orthosteric ligand binding site, is now understood to play a central role in receptor function and regulation.^10^ Interestingly, two structural nonatrienal variants, (2*E*,6*Z*)-nona-2,6-dienal and (2*Z*)-non-2-enal, were recently identified as positive allosteric modulators (PAMs), and the polycylic musk odorant, galaxolide, as negative allosteric modulator of human odorant receptor (OR) OR5AN1.^11^ Allosteric modulation has also been reported for insect odorant receptors,^12,13^ and for TAAR13c, a zebrafish olfactory receptor of the family of trace amine-associated receptors (TAARs).^14^

In humans, the detection of volatiles involves the chemoreception of odorants by ORs^15^ which are seven transmembrane domain G protein-coupled receptors (GPCRs),^16^ encoded by about 400 genes.^17,18^ ORs are typically located in the cilia of olfactory sensory neurons of the nose. They have evolved to best detect groups of ecologically relevant volatiles, such as key food odorants (KFOs) or semiochemicals.^19–22^

While the number of possible volatiles has been estimated in the billions,^23^ with more than 10,000 odorants known in food,^24^ the human odor space is still unknown.^25^ Those odorants, however, that appear in most foods in concentrations above their odor threshold and are necessary and sufficient to shape the odor quality of foods, by means of sensory testing of aroma recombinates and omission experiments, have been funneled down to about 230 KFOs.^20^ It is this group of experimentally tested odorants in the present work and previous publications that we refer to explicitly whenever using the terms ‘selective’ and ‘tuned’ for certain ORs.

A human receptor highly selective for sotolon among 187 KFOs has been identified and characterized recently.^26^ So far, however, an OR selective for (2*E*,4*E*,6*Z*)-nona-2,4,6-trienal has been unknown. Likewise, activation patterns of human ORs for food-typical recombinates^27^ have been largely elusive.

Here we set out to identify the best-responding human OR for (2*E*,4*E*,6*Z*)-nona-2,4,6-trienal, and to gain a mechanistic understanding of the receptor-based configurational coding of walnut odor at the receptor level.

Using GloSensor technology,^28^ we first screened our IL-6-HaloTag-OR cDNA library, comprising 766 human allelic variants,^21^ against (2*E*,4*E*,6*Z*)-nona-2,4,6-trienal. We then screened the responding receptor against 190 KFOs to identify its food-related ligand spectrum. Using flow cytometry, we confirmed OR surface expression in our HEK-293 test cell system. We finally established concentration–response relations for the single compounds, and demonstrated receptor allosterism of the walnut aroma recombinate at walnut-typical concentration ratios.

## Materials and Methods

### Chemicals

The following chemicals were used: D-luciferin (beetle) monosodium salt (Promega, Madison, USA); Pluronic PE-10500 (BASF, Ludwigs-hafen am Rhein, Germany); Dulbecco′s MEM medium, sotolon (3-hydroxy-4,5-dimethylfuran-2­(5*H*)-one, ≥97%; Merck, Darmstadt, Germany), and (2*E*,4*E*,6*Z*)-nona-2,4,6-trienal (>94%; aromaLAB, Martinsried, Germany).

The KFOs used were as published previously (Supporting Table S1).^20,21^ Further, structurally related substances were selected using the “Structure Search” tool from Sigma-Aldrich. These are butanal, pentanal, dodecanal, tridecanal, (2*E*)-but-2-enal, (2*E*)-pent-2-enal, (2*E*)-oct-2-enal, (6*Z*)-non-6-enal, (4*E*)-dec-4-enal, (2*E*)-dodec-2-enal, (2*E*)-tridec-2-enal, (2*E*,4*E*)-hexa-2,4-dienal, (2*E*,4*E*)-hepta-2,4-dienal, (2*E*,4*E*)-octa-2,4-dienal, (2*E*,6*E*)-nona-2,6-dienal, (2*E*,4*E*)-undeca-2,4-dienal (Merck, Darmstadt, Germany), (2*E*,4*E*)-dodeca-2,4-dienal (FisherScientific, Waltham, Massachusetts, USA) (Supporting Table S2). The absence of odor-active impurities in the reference odorants was checked by GC-O before their use.^29^

### Molecular Cloning of Human Odorant Receptors

All human ORs used in the present study were cloned and ligated into the expression plasmid IL-6-HaloTag-pFN210A, as described earlier,^30^ and were purified using the PureYield Plasmid Midiprep System (#A2495; Promega, Madison, USA) or the ZymoPURE II Plasmid Midiprep Kit (#D4201, Zymo Research, Irvine, USA).

### Cell Culture

We used HEK-293 cells,^31^ a human embryonic kidney cell line authenticated in 2021 (Eurofins genomics, Ebersberg, Germany), as a test cell system for the functional expression of recombinant ORs, as described earlier.^27,32^

### cAMP Luminescence Assay

Luminescence-based functional expression experiments were performed in HEK-293 cells using the pGloSensor-22F (Promega, Madison, USA), coding for a genetically engineered, cAMP-dependent luciferase. Transient transfection was performed using FuGENE 4K Transfection Reagent (#E4982; Promega, Madison, USA). For concentration–response experiments, each well was transfected with 50 ng OR plasmid, 10 ng Gαolf, 10 ng RTP1S, and 50 ng of the cAMP reporter pGloSensor-22F. The total DNA amount per well was 120 ng at a FuGENE:DNA ratio of 8:1.

For OR library screens, each well was transfected with 20 ng OR plasmid, 10 ng Gαolf, 10 ng RTP1S, and 10 ng pGloSensor-22F at a FuGENE:DNA ratio of 10:1. DMEM without FBS was used as serum-free medium. The transfection complexes were incubated for 15 min at room temperature and then added to the cells. Functional measurements were performed 42 h after transfection, quantifying odorant-induced, cAMP-dependent luminescence (RLU, relative luminescence units).

For receptor screening experiments we used a cDNA expression plasmid library comprising 766 cDNAs, coding for 386 human OR types (NCBI reference sequences) and 380 frequent variants as described previously.^30^ The cognate receptor–ligand combination OR1A1 and (5*R*)-2-methyl-5-(prop-1-en-2-yl)­cyclohex-2-en-1-one ((*R*)-carvone; 30 μmol/L) served as a positive control in all luminescence assays.

### Data Analysis of cAMP Luminescence Measurements

The raw luminescence data obtained from the Glomax MULTI+ detection system was analyzed as described previously.^32^

EC_50_ values and curves were derived from fitting the function

$$f\left(x\right)=\left(\frac{\left(\min-\max\right)}{\left({1+\left(\frac{x}{{EC}_{50}}\right)}^{Hillslope}\right)}\right)+\max$$

to the data by nonlinear regression (SigmaPlot 14.0, Systat Software, Inc., San Jose, CA, USA).

The data presented in this article was published with Mendeley (doi: 10.17632/rpncsfx5 fs.1).

### Flow Cytometry

HEK-293 cells were cultivated in 12-well plates (Corning Costar Flat Bottom Cell Culture Plates, #10136810, Corning Inc., NY, USA) with a density of 96,000 cells per well. On the next day, the transfection was performed using 400 ng OR plasmid, 80 ng Gαolf, 80 ng RTP1S, and 400 ng of the cAMP reporter pGloSensor-22F. The total DNA amount per well was 960 ng at a FuGENE:DNA ratio of 8:1. DMEM without FBS was used as serum-free medium. The transfection complexes were incubated for 15 min at room temperature and then added to the cells. Functional measurements were performed 42 h after transfection as described previously.^30^ Flow cytometry data were analyzed using FlowLogic software (version 7.3, Inivai, Mentone Victoria, Australia). A debris-exclusion gate (P2) was set on mock-transfected cells, and a positivity threshold (M2) was defined at ∼1% of the gated population. These gates were then applied identically to all receptor-transfected cells. The gating strategy is shown in Supporting Figure S1. One-way ANOVA was used for statistical analysis.

### Statistical Analyses

For statistical analysis and graphing we used Origin­(Pro) Version 2025 (OriginLab Corporation, Northampton, MA, USA) and SigmaPlot 14. We used the ShapiroWilk test as normality test. Differences between groups were evaluated using the Student’s *t*-test, the Mann–Whitney Rank Sum Test, or one-way ANOVA. The Median Absolute Deviation (MAD) threshold was calculated according to Chung et al. (2008),^33^ using the equation for non-normally distributed data:

MAD=median(|xi−median(x)|)

The “3× MAD” threshold employed in all screening experiments, therefore, represents the median +3× MAD.

## Results

### OR2J2 Solely Responded to (2*E*,4*E*,6*Z*)-Nona-2,4,6-trienal

We identified OR2J2 (and its haplotype OR2J2 V146A) as the sole responder to (2*E*,4*E*,6*Z*)-nona-2,4,6-trienal above a Median +3× Median Absolute Deviation (MAD) threshold out of 766 OR variants (Figure 1A).

**Figure 1:**
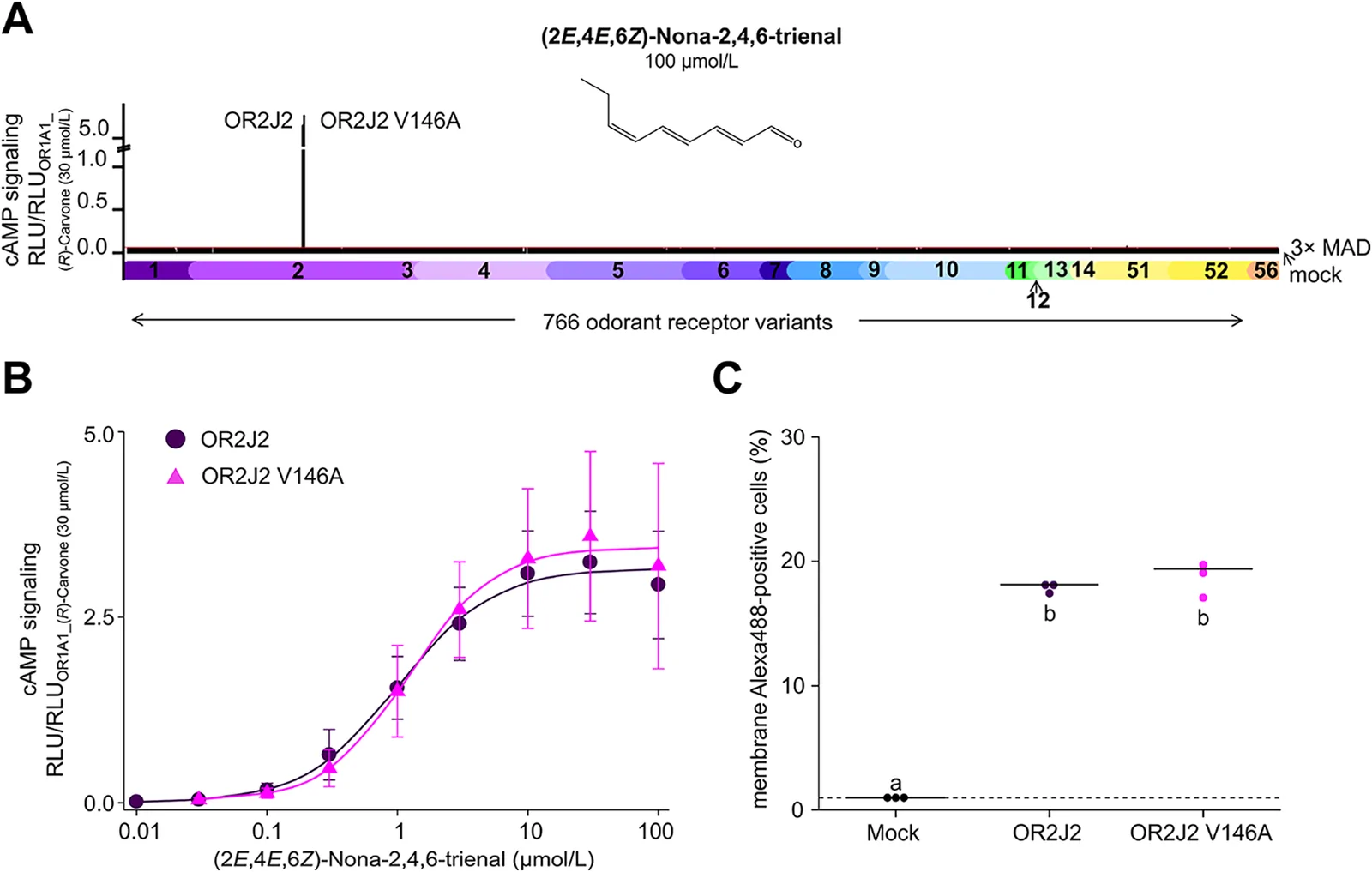
(2*E*,4*E*,6*Z*)-Nona-2,4,6-trienal selectively and concentration-dependently activates OR2J2 out of 766 OR variants. (A) Screening of 766 human OR variants with 100 μmol/L (2*E*,4*E*,6*Z*)-nona-2,4,6-trienal. Shown is the mean (*n* = 1 in duplicates). OR families are color-coded. Red line, 3× MAD threshold. Mock, empty plasmid control. RLU, relative luminescence units. (B) Concentration–response curves of (2*E*,4*E*,6*Z*)-nona-2,4,6-trienal on OR2J2 ref and OR2J2 V146A. Data are mean ± SD (OR2J2 ref: *n* = 8, OR2J2 V146A: *n* = 4). RLU, relative luminescence units. (C) Dot plot showing the surface expression of OR2J2 and its mutant OR2J2 V146A relative to mock. Data points represent individual measurements (*n* = 3). Black horizontal lines indicate the mean. The black dashed line indicates the mean of the mock control. Different letters indicate significant differences between groups (one-way ANOVA, Holm–Sidak method, *p* < 0.05). Both OR2J2 ref and OR2J2 V146A differed significantly from mock (*p* < 0.001), whereas OR2J2 V146A did not differ significantly from OR2J2 ref (*p* = 0.384).

(2*E*,4*E*,6*Z*)-Nona-2,4,6-trienal activated OR2J2 in a concentration-dependent manner with an EC_50_ value of 1.06 ± 0.15 μmol/L (Figure 1B, Table 1). OR2J2 V146A showed a comparable sensitivity toward (2*E*,4*E*,6*Z*)-nona-2,4,6-trienal, with an EC_50_ value of 1.17 ± 0.18 μmol/L, which did not differ significantly from the reference receptor (two-tailed Student’s *t-*test, *p* = 0.286). Although V146A displayed a slightly increased maximal response amplitude, this effect was not statistically significant (two-tailed Student’s *t*-test, *p* = 0.565). Similarly, the membrane expression of OR2J2 V146A was not significantly different from OR2J2 (Figure 1C).

**Table 1:** EC_50_ Values and Potencies of OR2J2 Agonists

chemical group^a^	no.^b^	compound	EC_50_ ± SD (μmol/L)^c^	potency (%)	KFO^d^
aliphatic aldehydes	1	(2*E*,4*E*,6*Z*)-nona-2,4,6-trienal	1.06 ± 0.15	100.00	√
aliphatic aldehydes	2	(2*E*,4*E*)-nona-2,4-dienal	8.94 ± 3.67	11.86	√
aliphatic aldehydes	3	(2*E*,4*E*)-undeca-2,4-dienal	9.66 ± 2.58	10.97	--
aliphatic aldehydes	4	(4*E*)-dec-4-enal	10.42 ± 0.55	10.17	--
aliphatic aldehydes	5	(2*E*,6*E*)-nona-2,6-dienal	10.94 ± 0.33	9.69	--
aliphatic aldehydes	6	(2*E*)-oct-2-enal	11.59 ± 1.77	9.15	--
aliphatic aldehydes	7	(2*E*)-dodec-2-enal	13.36 ± 2.36	7.93	--
aliphatic aldehydes	8	(4*Z*)-dec-4-enal	17.56 ± 5.31	6.04	√
aliphatic aldehydes	9	(2*E*)-undec-2-enal	18.52 ± 10.57	5.72	√
aliphatic aldehydes	10	(2*E*,4*E*)-octa-2,4-dienal	26.42 ± 5.43	4.01	--
aliphatic aldehydes	11	(2*E*,4*E*)-dodeca-2,4-dienal	28.92 ± 9.51	3.67	--
cinnamic acids and derivatives	12	ethyl cinnamate	28.95 ± 4.97	3.66	√
aliphatic aldehydes	13	octanal	30.71 ± 0.25	3.45	√
lactones	14	(5*R*/5*S*)-5-hexyloxolan-2-one	31.14 ± 4.22	3.40	√
coumarins and derivatives	15	7-methoxy-2*H*-1-benzopyran-2-one	33.15 ± 9.07	3.20	√
aliphatic aldehydes	16	(6*Z*)-non-6-enal	34.35 ± 0.96	3.09	--
aliphatic aldehydes	17	undecanal	37.86 ± 6.66	2.80	√
aliphatic aldehydes	18	(2*E*,4*E*)-hepta-2,4-dienal	38.41 ± 4.20	2.76	--
aliphatic aldehydes	19	(2*E*,4*E*)-deca-2,4-dienal	39.21 ± 6.88	2.70	√
aliphatic aldehydes	20	decanal	51.40 ± 11.50	2.06	√
fatty acyls	21	ethyl hexanoate	52.82 ± 20.80	2.01	√
phenols	22	2-methoxy-4-(prop-2-en-1-yl)phenol	53.33 ± 5.85	1.99	√
benzene and substituted derivatives	23	[(2*E*)-3-phenylprop-2-enyl] acetate	53.91 ± 2.43	1.97	√
aliphatic aldehydes	24	nonanal	57.90 ± 9.06	1.83	√
aliphatic aldehydes	25	(2*E*,6*Z*)-nona-2,6-dienal	65.81 ± 15.26	1.61	√
aliphatic aldehydes	26	(4*Z*)-hept-4-enal	74.72 ± 15.83	1.42	√
aliphatic aldehydes	27	(2*E*)-hept-2-enal	75.97 ± 25.84	1.40	√
aliphatic aldehydes	28	heptanal	89.07 ± 5.73	1.19	√
fatty acyls	29	(2*E*,6*Z*)-nona-2,6-dien-1-ol	91.22 ± 20.24	1.16	√
aliphatic aldehydes	30	hexanal	97.12 ± 21.62	1.09	√
aliphatic aldehydes	31	(2*E*,4*E*)-hexa-2,4-dienal	98.13 ± 9.65	1.08	--
benzene and substituted derivatives	32	1,2-dimethoxy-4-(prop-2-en-1-yl)benzene	112.22 ± 14.61	0.94	√
cinnamic acids and derivatives	33	methyl (2*E*)-3-phenylprop-2-enoate	124.50 ± 44.75	0.85	√
aliphatic aldehydes	34	(2*Z*)-oct-2-enal	139.72 ± 34.59	0.76	√
diazines	35	2,3-diethyl-5-methylpyrazine	174.86 ± 41.30	0.61	√
lactones	36	(5*R*/5*S*)-5-butyloxolan-2-one	185.73 ± 23.62	0.57	√
benzene and substituted derivatives	37	4-methoxybenzaldehyde	251.48 ± 25.40	0.42	√
aliphatic aldehydes	38	(2*E*)-dec-2-enal	255.13 ± 91.81	0.42	√
diazines	39	2-ethyl-3,5-dimethylpyrazine	421.99 ± 136.70	0.25	√
cinnamyl alcohols	40	[(2*E*)-3-phenylprop-2-enyl] acetate	589.16 ± 113.28	0.18	√
fatty acyls	41	hexan-1-ol	698.99 ± 56.24	0.15	√
aliphatic aldehydes	42	(2*E*)-non-2-enal	873.12 ± 213.91	0.12	√
phenols	43	3-methoxy-4-hydroxybenzaldehyde	975.70 ± 135.74	0.11	√
benzene and substituted derivatives	44	2-phenylethan-1-ol	1259.54 ± 652.97	0.08	√
fatty acyls	45	hexanoic acid	>1000	>0.10	√
aliphatic aldehydes	46	(2*Z*)-non-2-enal	4261.70 ± 451.51	0.02	√
aliphatic aldehydes	47	(3*Z*,6*Z*)-nona-3,6-dienal	4299.82 ± 447.57	0.02	√

a Chemical groups according to ClassyFire classification.^42^

b No., numbering according to the ranked order of EC_50_ values or potencies.

c EC_50_ values were derived from concentration–response curves of validated aldehyde agonists, which are shown in Supporting Figure S2. Graphs of compounds without a positive concentration-dependent effect are given in Supporting Figures S3, S4.

d KFO according to Dunkel et al. 2014.^20^

### KFO Screening Reveals a Pronounced Preference of OR2J2 for Aldehydes

OR2J2 has previously been linked to the KFOs 2*H*-1-benzopyran-2-one (coumarin), 3-methoxy-4-hydroxybenzaldehyde (vanillin), 3-ethoxy-4-hydroxybenzaldehyde (ethylvanillin) and nonanal, as well as 12 additional agonists,^34–38^ suggesting a broader ligand spectrum. Thus, we aimed to establish a comprehensive, food-related, EC_50_-ranked KFO agonist profile of OR2J2, and to find out whether this receptor nevertheless exhibits a selectivity for a single compound, e.g., (2*E*,4*E*,6*Z*)-nona-2,4,6-trienal. This is not an unusual feature of ORs: Previously, we identified OR1A1 to be rather broadly tuned, responding to 23 out of 202 tested compounds (17 out of 190 KFOs). However, OR1A1 turned out to be selective for the KFO 3-methylnonane-2,4-dione, displaying a potency that stood out 2 orders of magnitude compared to all other KFOs and non-KFOs tested.^38–40^ KFOs have been proposed as natural agonists for many ORs.^20,41^ We, therefore, focused on those food-related compounds initially published by Dunkel et al. (2014) to characterize the KFO agonist spectrum of OR2J2.^20^

Screening of 190 KFOs from 29 chemical groups at a concentration of 300 μmol/L against OR2J2 identified 27 true positive agonists (Figure 2, Supporting Figure S2).

**Figure 2:**
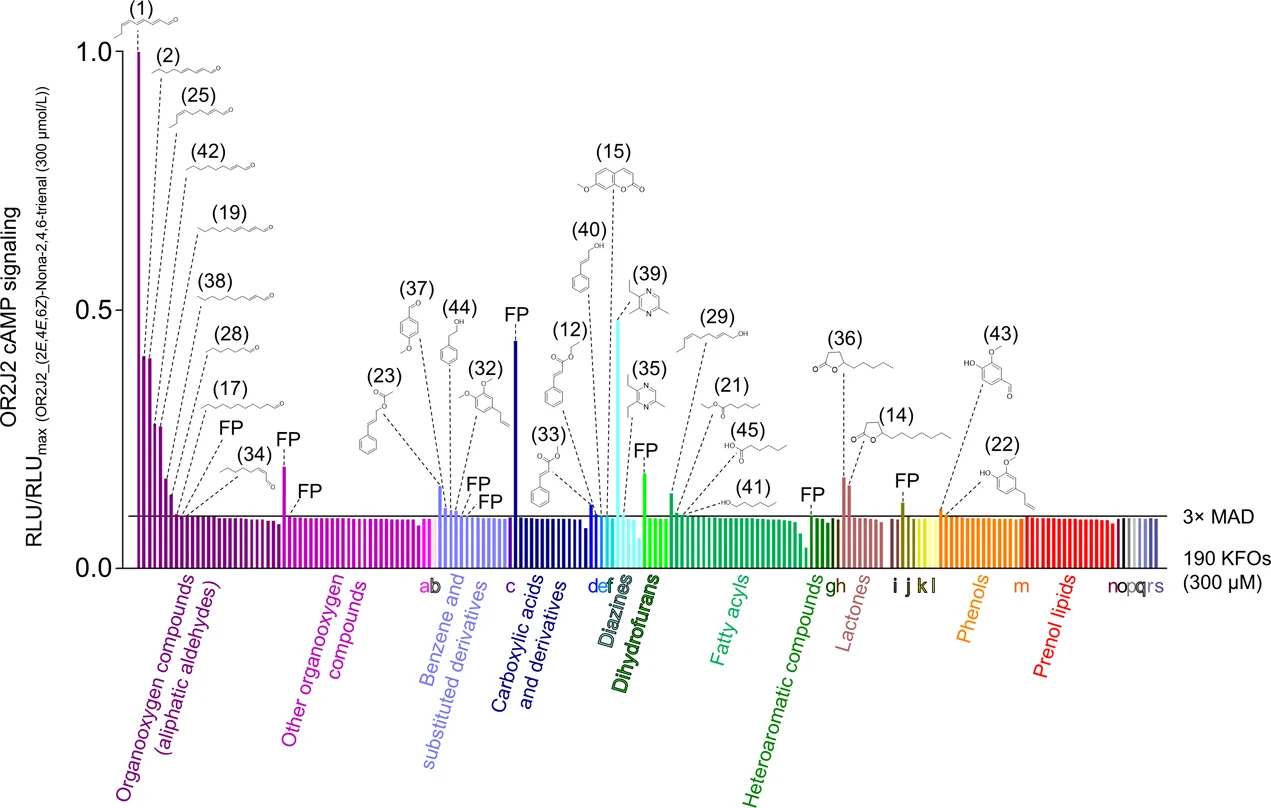
OR2J2 is tuned to aliphatic aldehydes among KFOs. Twenty-seven agonists emerged from testing 190 KFOs at 300 μmol/L against OR2J2. Data are mock-subtracted and given as mean (*n* = 1, measured in triplicates), normalized to the maximum response. Numbers are according to the order of potencies in Table 1. Black line, 3× MAD threshold; RLU, relative luminescence units. FP, false positives. KFOs are grouped according to the ClassyFire classification.^42^ a = allyl sulfur compounds, b = azolines, c = benzodioxoles, d = cinnamic acids and derivatives, e = cinnamyl alcohols, f = coumarines and derivatives, g = hydroxy acids and derivatives, h = indoles and derivatives, i = organic disulfides, j = organic trisulfides, k = oxanes, l = phenol ethers, m = phenylpropanoic acids, n = pyrans, o = pyridins and derivatives, p = pyrrolines, q = thioacetals, r = thioethers, s = thiols.

Notably, hits were concentrated in a limited number of chemical classes, rather than being broadly distributed across the KFO library. Aliphatic aldehydes contributed the largest subclass of agonists, and included two of the three most potent ligands. Additional agonists were identified among benzene and substituted derivatives, cinnamic acid derivatives, coumarins, diazines, fatty acyls, lactones, and phenols, whereas many other chemical classes represented in the KFO library did not yield validated agonists. Thus, the primary screen suggested a pronounced, though not exclusive, preference of OR2J2 for aldehydic odorants. Concentration–response curves of validated agonists are shown in Supporting Figure S2, those of false positive KFO screening hits in Supporting Figure S3.

### OR2J2 Activation is Shaped by Chain Length and Degree of Unsaturation

Prompted by the preference for aldehydes of OR2J2 in the KFO screen, we next performed a dedicated aldehyde screen spanning different chain lengths and different degrees of unsaturation, including additional non-KFO aldehydes (Figure 3).

**Figure 3:**
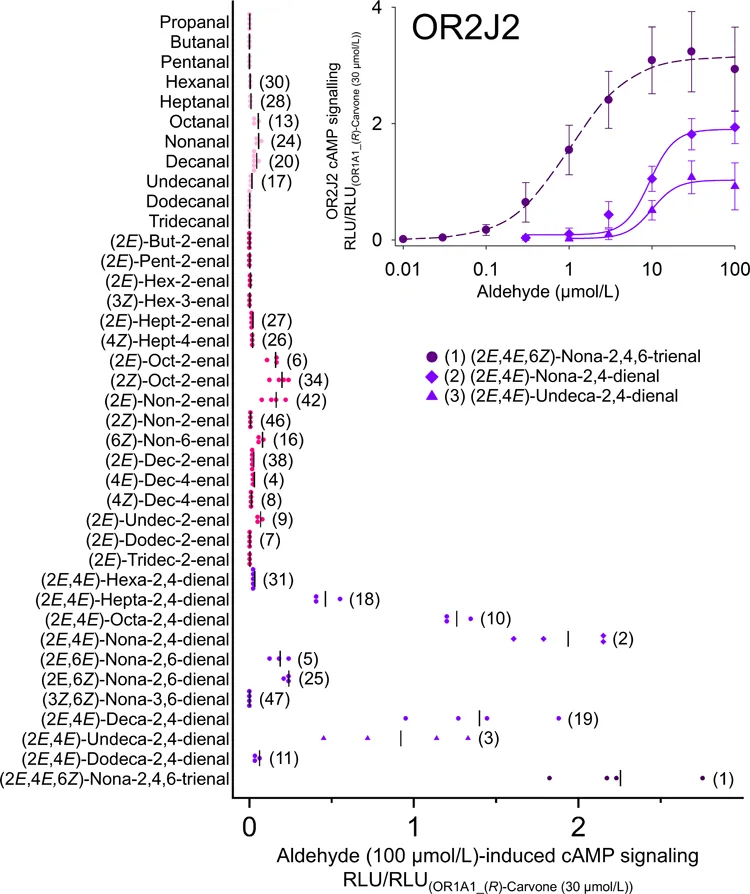
OR2J2 is selective for medium-chain conjugated dienals and (2*E*,4*E*,6*Z*)-nona-2,4,6-trienal. Aldehyde screen of 39 compounds tested at 100 μM against OR2J2. Each data point represents an individual measurement; the black vertical lines indicate the mean. Numbers indicate the ascending order of EC_50_ values (Table 1). Color coding indicates the degree of unsaturation. Compounds without numbers are false positives. **Insert:** concentration–response curves of the most potent agonists identified in the screen. The (2*E*,4*E*,6*Z*)-nona-2,4,6-trienal curve (dashed line) was taken from Figure 1B. Data are shown as mean ± SD (*n* = 4–8). All data were normalized to the response of OR1A1 to 30 μM (*R*)-carvone.

The screen revealed a clear preference of OR2J2 for medium-chain unsaturated aldehydes. In general, saturated aldehydes showed lower potencies (higher EC_50_ values) and lower maximal responses than unsaturated analogs, whereas monounsaturated aldehydes displayed more heterogeneous activities. The highest potencies and efficacies were observed for conjugated dienals and, most prominently, for (2*E*,4*E*,6*Z*)-nona-2,4,6-trienal.

Concentration–response analyses of the three most potent agonists, (2*E*,4*E*,6*Z*)-nona-2,4,6-trienal, (2*E*,4*E*)-nona-2,4-dienal, and (2*E*,4*E*)-undeca-2,4-dienal, confirmed their strong activation of OR2J2 (Figure 3, Table 1), and suggested a unique position of (2*E*,4*E*,6*Z*)-nona-2,4,6-trienal within the OR2J2 agonist spectrum, displaying a 6-fold lower EC_50_ than the second best agonists.

### (2*E*,4*E*,6*Z*)-Nona-2,4,6-trienal Outperforms the Potencies of 46 Other OR2J2 Agonists

To place this exceptional potency into the context of all validated OR2J2 agonists, agonist-space analysis based on all validated agonists from the KFO and aldehyde screens further illustrated the exceptional position of (2*E*,4*E*,6*Z*)-nona-2,4,6-trienal within the OR2J2 ligand spectrum (Figure 4). In both the KFO-derived (Figure 4A) and aldehyde-focused (Figure 4B) agonist spaces, (2*E*,4*E*,6*Z*)-nona-2,4,6-trienal was the only ligand assigned to the highest-potency range (EC_50_ < 3 μmol/L), whereas all other agonists fell into lower potency ranges.

**Figure 4:**
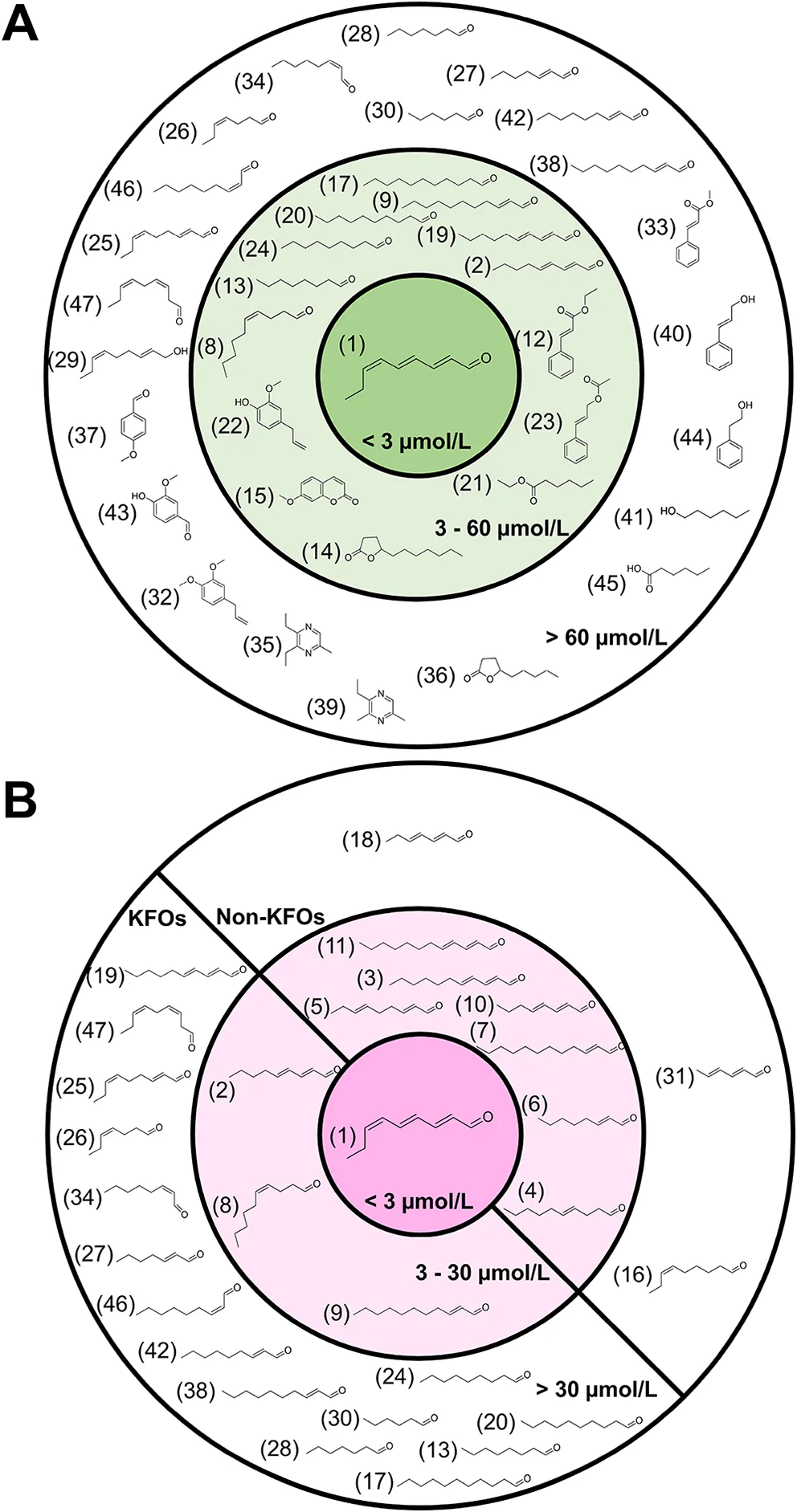
Agonist space of OR2J2 highlights (2*E*,4*E*,6*Z*)-nona-2,4,6-trienal as the most potent ligand. (A) KFO agonist space of OR2J2. (2*E*,4*E*,6*Z*)-Nona-2,4,6-trienal was the only KFO exhibiting an EC_50_ below 3 μmol/L. All other agonists were assigned to the outer range (≥3 μmol/L). (B) Aldehyde agonist space of OR2J2. (2*E*,4*E*,6*Z*)-Nona-2,4,6-trienal was the only aldehyde exhibiting an EC_50_ below 3 μmol/L. All other agonists were assigned to the outer range (≥3 μmol/L). Numbers indicate ranked order of potencies (Table 1).

### Walnut Odorants Reveal Noncompetitive, Allosteric Modulation at OR8D1

Because (2*E*,4*E*,6*Z*)-nona-2,4,6-trienal emerged as the most potent OR2J2 agonist among 203 tested compounds and co-occurs with sotolon as characteristic KFOs in walnuts, we next asked whether these two odorants interact at the receptor level. To this end, we screened our OR library with a walnut recombinate containing food-grade concentrations of (2*E*,4*E*,6*Z*)-nona-2,4,6-trienal and sotolon (10 μg/kg each; 1:1; Supporting Figure S5).^2^ Under these conditions, no receptor activation was detected, likely because these concentrations were too low for the in vitro assay. However, Stübner and Steinhaus (2023) showed that 100 μg/kg each of (2*E*,4*E*,6*Z*)-nona-2,4,6-trienal and sotolon intensifies walnut aroma, prompting us to investigate receptor responses at higher concentrations.^2^ Again, solely OR2J2 was activated above a 3× MAD-threshold (Supporting Figure S6). At a concentration of 0.78 μmol/L, sotolon did not yet induce a measurable activation of OR8D1. We therefore established concentration–response relations for OR8D1. Conversely, we tested whether sotolon activates OR2J2 or (2*E*,4*E*,6*Z*)-nona-2,4,6-trienal activates OR8D1, and whether these ligands interact at the receptor level (Figure 5). Neither receptor, however, responded to the respective noncognate ligand.

**Figure 5:**
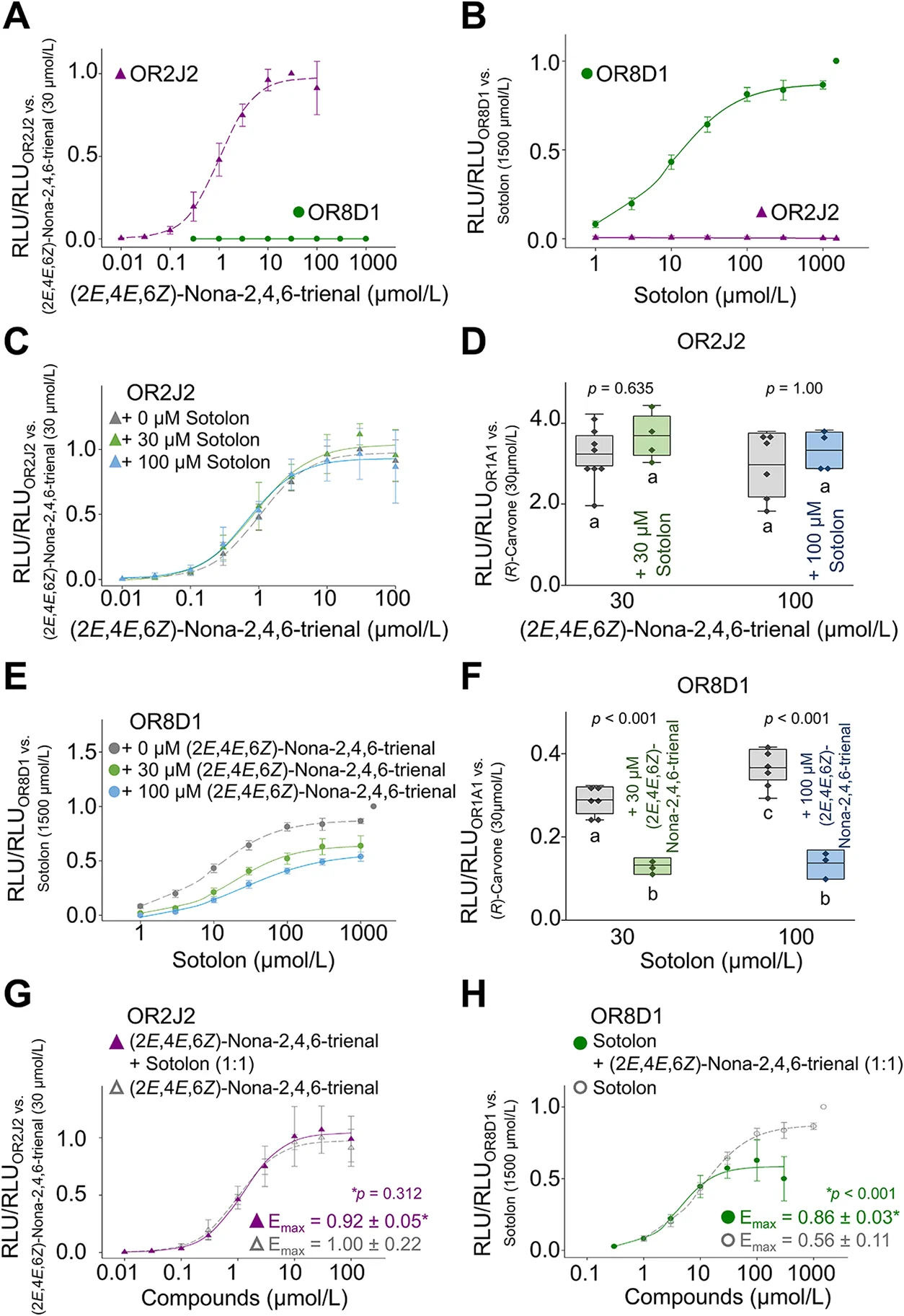
(2*E*,4*E*,6*Z*)-Nona-2,4,6-trienal allosterically modulates OR8D1. Unless stated otherwise, data were mock control-subtracted and are displayed as mean ± SD. The (2*E*,4*E*,6*Z*)-nona-2,4,6-trienal concentration–response curve for OR2J2 (dashed line) was taken from Figure 1B and is shown in (A), (C), and (G) for comparison; the sotolon curve from (B, solid line) is reproduced in (E) and (H) (dashed line). (A) Concentration–response relations of (2*E*,4*E*,6*Z*)-nona-2,4,6-trienal on OR2J2 (purple triangles) and OR8D1 (green circles), normalized to OR2J2 response to (2*E*,4*E*,6*Z*)-nona-2,4,6-trienal (30 μmol/L; *n* = 3–8). (B) Concentration–response relations of sotolon on OR8D1 (green circles) and OR2J2 (purple triangles), normalized to OR8D1 response to sotolon (1500 μmol/L; *n* = 3–6). (C) Modulation of OR2J2 activation by (2*E*,4*E*,6*Z*)-nona-2,4,6-trienal in the presence of sotolon (0, 30, 100 μmol/L); normalization as in (A) (*n* = 4–8). (D) OR2J2 activation by (2*E*,4*E*,6*Z*)-nona-2,4,6-trienal (30 and 100 μmol/L) with or without sotolon cotreatment (one-way ANOVA, *p* = 0.34). Data normalized to OR1A1 response to (*R*)-carvone (30 μmol/L) and shown as box-and-whisker plots; boxes, 25th -75th percentiles; whiskers, full data range; horizontal lines, means (*n* = 4–8). Different letters denote significant group differences. (E) Modulation of OR8D1 activation by sotolon in the presence of (2*E*,4*E*,6*Z*)-nona-2,4,6-trienal (0, 30, 100 μmol/L); normalization as in (B) (*n* = 3–6). (F) OR8D1 activation by sotolon is significantly reduced by (2*E*,4*E*,6*Z*)-nona-2,4,6-trienal cotreatment at both 30 and 100 μmol/L (one-way ANOVA, Holm–Sidak, *p* < 0.05). Normalization and display as in (D) (*n* = 3–6). (G) A 1:1 mixture of (2*E*,4*E*,6*Z*)-nona-2,4,6-trienal and sotolon (filled purple triangles) did not significantly alter OR2J2 activation compared with (2*E*,4*E*,6*Z*)-nona-2,4,6-trienal alone (*E*_max_, two-sided *t* test, *p* = 0.31); normalization as in (A) (*n* = 5–8). (H) The 1:1 mixture (filled green circles) significantly reduced OR8D1 activation compared with sotolon alone (*E*_max_, two-sided *t* test, *p* = 0.00022); normalization as in (B) (*n* = 4–6). RLU, relative luminescence units; SD, standard deviation.

(2*E*,4*E*,6*Z*)-Nona-2,4,6-trienal activated OR2J2, but not OR8D1, in a concentration-dependent manner (Figure 5A). Conversely, sotolon, but not (2*E*,4*E*,6*Z*)-nona-2,4,6-trienal, induced a concentration-dependent activation of OR8D1 (Figure 5B).

We next examined whether the two walnut odorants modulate each other’s cognate receptors. The presence of sotolon at concentrations of 30 or 100 μmol/L did not measurably affect (2*E*,4*E*,6*Z*)-nona-2,4,6-trienal’s maximum efficacy (*E*_max_ at 30 and 100 μmol/L) in activating OR2J2 (Figure 5C,D). The (2*E*,4*E*,6*Z*)-nona-2,4,6-trienal concentration–response relation on OR2J2 did not change in the presence of 100 μmol/L sotolon (Figure 5C). The EC_50_, however, decreased 1.8-fold under 30 μmol/L sotolon (Table 2). Vice versa, and in contrast, the presence of (2*E*,4*E*,6*Z*)-nona-2,4,6-trienal at concentrations of 30 or 100 μmol/L significantly attenuated the sotolon-induced activation of OR8D1, with an about 2-fold increase in EC_50_, and a significantly reduced efficacy (Figure 5E,F, Table 2).

**Table 2:** EC_50_ and *E*_max_ Values for OR2J2 and OR8D1 in Response to (2*E*,4*E*,6*Z*)-Nona-2,4,6-trienal, Sotolon, and their Binary Mixtures^a^,^b^ ^,^ ^b^

OR2J2	EC_50_ (μmol/L)	*E*_max_	*n*
(2*E*,4*E*,6*Z*)-nona-2,4,6-trienal	1.06 ± 0.15 a	0.92 ± 0.05 a	8
(2*E*,4*E*,6*Z*)-nona-2,4,6-trienal + 30 μmol/L sotolon	0.94 ± 0.13 a	0.90 ± 0.19 a	4
(2*E*,4*E*,6*Z*)-nona-2,4,6-trienal + 100 μmol/L sotolon	0.99 ± 0.27 a	0.97 ± 0.12 a	4
(2*E*,4*E*,6*Z*)-nona-2,4,6-trienal + sotolon (1:1)	1.78 ± 0.84 a	1.00 ± 0.22 a	5

OR8D1	EC_50_ (μmol/L)	*E*_max_	*n*
sotolon	15.33 ± 5.99 a	0.86 ± 0.03 a	6
sotolon + 30 μmol/L (2*E*,4*E*,6*Z*)-nona-2,4,6-trienal	20.95 ± 5.85 ab	0.59 ± 0.08 b	3
sotolon + 100 μmol/L (2*E*,4*E*,6*Z*)-nona-2,4,6-trienal	26.43 ± 4.87 b	0.48 ± 0.04 b	3
sotolon + (2*E*,4*E*,6*Z*)-nona-2,4,6-trienal (1:1)	4.78 ± 1.90 c	0.56 ± 0.11 b	4

a OR2J2 EC_50_ and *E*_max_: Because normality was not met (Shapiro–Wilk test, *p* < 0.05), group differences were analyzed using a Kruskal–Wallis one-way analysis of variance on ranks. No significant differences were observed for EC_50_ (*p* = 0.21) or *E*_max_ (*p* = 0.54).

b OR8D1 EC_50_ and *E*_max_: Different letters indicate significant differences between groups (one-way ANOVA, Holm–Sidak method, *p* < 0.05).

For each receptor, we then compared the concentration–response relation of its cognate agonist with that of the binary mixture, keeping the concentration ratio of 1:1 constant throughout. On OR2J2, the concentration–response curve of the mixture was essentially identical with that of (2*E*,4*E*,6*Z*)-nona-2,4,6-trienal alone, lacking any effect on potency or efficacy (Figure 5G, Table 2). Accordingly, the concentration–response-curves of haplotype OR2J2 V146A for (2*E*,4*E*,6*Z*)-nona-2,4,6-trienal and for the 1:1 mixture with sotolon did not differ, and OR2J2 V146A could not be activated by sotolon alone (Supporting Figure S7). On OR8D1, however, the concentration–response relation of the binary mixture showed a 35% reduction in efficacy and a 3-fold lower EC_50_ compared to sotolon alone (Figure 5H, Table 2), but without altering threshold concentrations.

## Discussion

Here, we newly identified OR2J2 being tuned to medium-chain unsaturated aldehydes with a selectivity for (2*E*,4*E*,6*Z*)-nona-2,4,6-trienal. Nona-2,4,6-trienals have been detected in a wide array of foods. (2*E*,4*E*,6*Z*)-Nona-2,4,6-trienal is one of the most odor-active food volatiles, recognized as key food odorant in walnuts,^2^ oat flakes,^43^ and black tea.^44^ Aldehydes, but not (2*E*,4*E*,6*Z*)-nona-2,4,6-trienal, are included in the KFO-centered agonist spectra of the human receptors OR1A1 and OR2W1 (16 and 71 agonists, out of 190 and 187 tested KFOs, for OR1A1 and OR2W1, respectively).^39,40^ However, only a few ORs have been identified to respond selectively to aldehydes.^45,46^ OR10A6 L287P, for example, was described as a human receptor responsive to the unsaturated aldehydes (4*Z*)-undec-4-enal, (4*Z*)-non-4-enal, and (6*Z*)-undec-6-enal, whereas OR2W1 also responded to these ligands, albeit at higher threshold concentrations.^46^ Similarly, our KFO and aldehyde screens indicate a pronounced, though not exclusive, preference of OR2J2 for aldehydic ligands. This is corroborated by previous deorphanization studies linking OR2J2 not only to 2*H*-1-benzopyran-2-one (coumarin) and nonanal, but also to several medium-chain aliphatic alcohols, including heptan-1-ol, octan-1-ol, nonan-1-ol, and decan-1-ol.^34,36,37^ However, in our study, aliphatic aldehydes were enriched among the validated agonists: 70% of the aliphatic aldehydes in the screened KFO library activated the receptor, versus 31% KFOs of the next most responsive chemical class (benzenes and substituted derivatives). In summary, we characterized OR2J2 as the sole (2*E*,4*E*,6*Z*)-nona-2,4,6-trienal-responsive receptor out of a screen of 766 human OR variants, with (i) a preference for medium-chain unsaturated aldehydes over saturated aldehydes, (ii) with conjugated dienals among its best agonists, and (iii) with a selectivity for (2*E*,4*E*,6*Z*)-nona-2,4,6-trienal as the most potent and efficient KFO agonist, so far. Our findings are corroborated by a previous report demonstrating that aldehyde-responsive receptors can have broadly or narrowly tuned KFO agonist profiles, and that unsaturated aldehydes may elicit stronger responses than their saturated analogs.^47^ Together, our findings strongly suggest OR2J2 as a key receptor participating in the perception of walnut aroma.

It must be pointed out that we may have missed other (2*E*,4*E*,6*Z*)-nona-2,4,6-trienal-responsive ORs or responsive genetic OR variants, since (i) our receptor screening experiments did not include all known genetic human OR variants, and (ii) some receptors from our collection may not work with the assay used in our study. We did not further investigate putative inverse agonists (Supporting Figure S3D,G).

Beyond their role as human KFOs, odorants may function as ecologically relevant semiochemicals in a species-specific context.^21,22,48,49^ Among these semiochemicals, reviews and online databases list a wide structural variety of aldehydes, including dienals and trienals.^50,51^ For example, nona-2,4,6-trienal isomers have been reported as insect pheromones and were identified from the male tobacco and eggplant flea beetles, *Epitrix hirtipennis* and *E. fuscula*.^52^ (2*E*,6*Z*)-Nona-2,6-dienal and (2*E*)-non-2-enal are known as male sex pheromone in the scorpionfly *Panorpa germanica* ^53^ and in the melonfly *Zeugodacus cucurbitae*.^54^ In addition, the exact same two volatile aldehydes have been reported to be solely emitted by damaged barley roots.^55^ Since insect ORs are heteromeric ion channels, which differ profoundly in structure from vertebrate ORs, the selective detection of nona-2,4,6-trienals across Phyla, thus, suggests evolutionary convergence.

OR8D1 was identified as a sotolon-selective receptor, with an EC_50_ value in the low micromolar range,^26^ which has been confirmed in a recent study.^56^ (2*E*,4*E*,6*Z*)-Nona-2,4,6-trienal up to millimolar concentration did not activate OR8D1 orthosterically, but rather modulated the sotolon function at OR8D1 by attenuating its efficacy, both at single (2*E*,4*E*,6*Z*)-nona-2,4,6-trienal concentration, or in the concentration–response relation of the (2*E*,4*E*,6*Z*)-nona-2,4,6-trienal-sotolon 1:1 walnut recombinate. Under the latter experimental condition, importantly, no change in threshold concentration occur-red — (2*E*,4*E*,6*Z*)-nona-2,4,6-trienal exclusively affected sotolon’s efficacy in activating OR8D1, strongly suggesting (2*E*,4*E*,6*Z*)-nona-2,4,6-trienal as a negative allosteric modulator of OR8D1.

Altogether, our results suggest OR2J2 and OR8D1 as key receptors being orthosterically activated by the single components (2*E*,4*E*,6*Z*)-nona-2,4,6-trienal and sotolon, respectively. As a 1:1 mixture, both compounds encode the configurational walnut aroma,^2^ with (2*E*,4*E*,6*Z*)-nona-2,4,6-trienal additionally acting as a negative allosteric modulator at OR8D1.

Our findings elucidate the molecular basis of walnut aroma, and underscore the importance of receptor allosterism in odor coding. Since a single OR may be activated by several odorants, negative allosteric modulation of OR activity by odorants in complex mixtures, therefore, may prevent saturation of OR activity and enable quantitative or analytical coding in addition to combinatorial coding.^7^ Allosteric modulation, thus, may sharpen the contrast of OR activity patterns for odorant mixtures.^11^

In summary, our findings support the notion that ORs are multidimensional coincidence detectors, such that configurational food odor identities are immanently encoded by distinct, food-typical odorant concentration ratios, with orthosteric and allosteric functions at the receptor level. Further research is needed to explore (i) the broader implications of these interactions in olfactory food perception, and (ii) the potential for leveraging allosteric receptor–ligand interactions to modulate sensory profiles of food products.

## Supplementary Material



## Notes

Experimental details on investigated odorants and aldehydes, flow cytometry gating strategy, odorant concentration–response relations on OR2J2, validation of false-positive screening signals, OR library screening of walnut recombinates, and functional analysis of OR2J2 haplotype V146A (PDF)

## Abbreviations used

GPCR: G protein-coupled receptor
KFO: key food odorant
OR: odorant receptor
PAM: positive allosteric modulator
TAAR: trace amine-associated receptor
