# Supplementary material for: Receptor Allosterism Governs the Coding of Configurational Walnut Aroma
Source: J Agric Food Chem. 2026 Oct 29;74(44):35048–57. doi: 10.1021/acs.jafc.6c10400 (PMC13761274; doi:10.1021/acs.jafc.6c10400)
Supplement: Supplementary file 1 [file jf6c10400_si_001.pdf]

Supporting Information

Receptor allosterism governs the coding of configurational walnut aroma

Ronja Hopf,[1,2#] Franziska Haag,[2#] Stephanie Frank,[2] Sandra Hoffmann,[2] Martin Steinhaus,[2] and Dietmar Krautwurst[2*]

[1]TUM School of Life Sciences, Technical University of Munich, 85354 Freising, Germany

[2]Leibniz Institute for Food Systems Biology at the Technical University of Munich, 85354 Freising, Germany

[#]These authors contributed equally

[*]Correspondence: d.krautwurst.leibniz-lsb@tum.de

Contents:

Table S1. Investigated key food odorants according to Dunkel et al. (2014)[1] 2

Table S2. Additionally investigated aldehydes. 10

Figure S1. Gating strategy for cell surface expression analyses. 11

Figure S2. Concentration-response-relations of aldehyde and/or KFO agonists on OR2J2. 12

Figure S3. Validation of false-positive KFO hits on OR2J2. 16

Figure S4. Certain aldehydes failed to activate OR2J2. 17

Figure S5. No receptor activation measurable at food grade concentrations.....................18

Figure S6. Activation pattern of walnut recombinate at 10× food grade concentrations. ..18

Figure S7. OR2J2 haplotype V146A is not activated or modulated by sotolon...................19

Table S1. Investigated key food odorants according to Dunkel et al. (2014)[1]

key food odorant	CAS#
(1*R*,5*R*)-6,6-dimethyl-2-methylenebicyclo[3.1.1]heptane ((+)-β-pinene)	19902-08-0
(1R,5R)-2,6,6-trimethylbicyclo[3.1.1]hept-2-ene ((+)-α-Pinene)	7785-70-8
(1*R*/1*S*)-1,3,3-trimethylbicyclo[2.2.1]heptan-2-one (fenchone), sum of isomers	1195-79-5
(1*S*,5*S*)-1-isopropyl-4-methylenebicyclo[3.1.0]hexane ((+)-sabinene)	2009-00-9
(1S,5S)-2,6,6-trimethylbicyclo[3.1.1]hept-2-ene ((−)-α-Pinene)	7785-26-4
(1*S*,5*S*)-6,6-dimethyl-2-methylenebicyclo[3.1.1]heptane ((−)-β-pinene)	18172-67-3
(2*E*)-1-(2,6,6-trimethylcyclohex-1-en-1-yl)but-2-en-1-one ((*E*)-β-damascone)	23726-91-2
(2*E*)-1-(2,6,6-trimethylcyclohexa-1,3-dien-1-yl)but-2-en-1-one ((*E*)-β-damascenone)	23726-934
(2*E*)-3,7-dimethylocta-2,6-dien-1-ol (geraniol)	106-24-1
(2*E*)-3-phenylprop-2-en-1-ol (cinnamyl alcohol)	104-54-1
(2*E*)-dec-2-enal	3913-81-3
(2*E*)-hept-2-enal	18829-55-5
(2*E*)-hex-2-enal	6728-26-3
(2*E*)-non-2-enal	18829-56-6
(2*E*)-undec-2-enal	53448-07-0
(2*E*,4*E*)-deca-2,4-dienal	25152-84-5
(2*E*,4*E*)-nona-2,4-dienal	5910-87-2
(2*E*,4*E*,6*Z*)-nona-2,4,6-trienal	100113-52-8
(2*E*,5*R*/5*S*)-5-methylhept-2-en-4-one (filbertone), sum of isomers	102322-83-8
(2*E*,6*Z*)-nona-2,6-dien-1-ol	28069-72-9
(2*E*,6*Z*)-nona-2,6-dienal	26370-28-5
(2*R*/2*S*)-2-methylbutan-1-ol, sum of isomers	137-32-6
(2*R*/2*S*)-2-methylbutyl acetate, sum of isomers	624-41-9
(2*R*/2*S*)-2-sulfanylpetan-3-one, sum of isomers	17042-24-9
(2*R*/2*S*)-4-methoxy-2,5-dimethylfuran-3(2*H*)-one, sum of isomers	4077-47-8

Table S1: Investigated key food odorants according to Dunkel et al. (2014)[1] (continued).

key food odorant	CAS#
(2*R*/2*S*,3*R*/3*S*)-2-methyl-3-sulfanylpentan-1-ol, sum of isomers	227456-27-1
(2*S*,4*R*)/(2*R*,4*S*)-4-methyl-2-(2-methylprop-1-en-1-yl)tetrahydro-2*H*-pyran ((+/-)-*cis*-rose oxide) sum of isomers	16409-43-1
(2*Z*)-non-2-enal	60784-31-8
(2*Z*)-oct-2-enal	20664-46-4
(3*E*)-4-(2,6,6-trimethylcyclohex-1-en-1-yl)but-3-en-2-one (β-ionone)	79-77-6
(3*R*/3*S*)-3-hydroxybutan-2-one (acetoin), sum of isomers	513-86-0
(3*R*/3*S*)-3-methylnonane-2,4-dione, sum of isomers	113486-29-6
(3*R*/3*S*)-3-sulfanylpentan-2-one, sum of isomers	67633-97-0
(3*R*/3*S*)-oct-1-en-3-ol, sum of isomers	3391-86-4
(3*R*/3*S*)-3,7-dimethylocta-1,6-dien-3-ol ((*R*/*S*)-linalool), sum of isomers	78-70-6
(3*Z*)-hex-3-en-1-ol	928-96-1
(3*Z*)-hex-3-en-1-yl acetate	3681-71-8
(3*Z*)-hex-3-enal	6789-80-6
(3*Z*,6*Z*)-nona-3,6-dienal	21944-83-2
(4*R*/4*S*)-1-methyl-4-(prop-1-en-2-yl)cyclohex-1-ene ((*R*/*S*)-limonene), sum of isomers	138-86-3
(4*R*/4*S*)-4-methyloctanoic acid, sum of isomers	54947-74-9
(4*S*)-1-methyl-4-(prop-1-en-2-yl)cyclohex-1-ene ((*S*)-(−)-limonene)	5989-54-8
(4*S*,5*S*)-5-butyl-4-methyldihydrofuran-2(3*H*)-one ((3*S*,4*S*)-*cis*-whiskylactone), sum of isomers	55013-32-6
(4*Z*)-dec-4-enal	21662-09-9
(4*Z*)-hept-4-enal	6728-31-0
(5*R*)-2-methyl-5-(prop-1-en-2-yl)cyclohex-2-en-1-one ((*R*)-(−)-carvone)	6485-40-1
(5*R*/5*S*)-5-butyloxolan-2-one ((4*R*/4*S*))-γ-nonalactone, sum of isomers	104-61-0
(5*R*/5*S*)-5-ethyl-3-hydroxy-4-methylfuran-2(5*H*)-one (abhexon), sum of isomers	698-10-2

Table S1: Investigated key food odorants according to Dunkel et al. (2014)[1] (continued).

key food odorant	CAS#
(5*R*/5*S*)-5-heptyloxolan-2-one ((4*R*/4*S*)-γ-dodecalactone), sum of isomers	706 14 9
(5*R*/5*S*)-5-hexyloxolan-2-one ((4*R*/4*S*)-γ-decalactone), sum of isomers	706-14-9
(5*R*/5*S*)-5-hexyloxolan-2-one ((4*R*/4*S*)-γ-undecalactone), sum of isomers	104-67-6
(5*R*/5*S*)-5-propyloxolan-2-one ((4*R*/4*S*)-γ-octalactone), sum of isomers	104-50-7
(5*S*)-2-methyl-5-(propan-2-yl)cyclohexa-1,3-diene ((*R*)-α-phellandrene)	4221-98-1
(5*Z*)-octa-1,5-dien-3-one	65767-22-8
(6*R*/6*S*)-6-heptyloxan-2-one ((5*R*/5*S*)-δ-dodecalactone), sum of isomers	713-95-1
(6*R*/6*S*)-6-hexyloxolan-2-one ((5R)/(5S)-δ-decalactone)	705-86-2
(6*R*/6*S*)-6-propyloxan-2-one ((5*R*/5*S*)-δ-octalactone), sum of isomers	698-76-0
(methyldisulfanyl)methane (dimethyl disulfide)	624-92-0
(methylsulfanyl)methane (dimethyl sulfide)	75-18-3
[(2*E*)-3-phenylprop-2-enyl] acetate (cinnamyl acetate)	103-54-8
1-(3,4-dihydro-2*H*-pyrrol-5-yl)propan-1-one	133447-37-7
1(4-methylphenyl)ethan-1-one	122-00-9
1-(propyldisulfanyl)propane (dipropyl disulfide)	629-19-6
1-(pyrazin-2-yl)ethan-1-one	22047-25-2
1,1-diethoxyethane (acetal)	105-57-7
1,2-dimethoxy-4-(prop-2-en-1-yl)benzene (methyl eugenol)	93-15-2
1,3,3-trimethyl-2-oxabicyclo[2.2.2]octane (1,8-cineole)	470-82-6
1,3-benzodioxole-5-carbaldehyde (piperonal)	120-57-0
12-methyltridecanal	75853-49-5
1-methoxy-4-(prop-2-en-1-yl)benzene (estragole)	140-67-0
1-methoxy-4-[(*E*)-prop-1-en-1-yl]benzene (*trans*-anethole)	4180-23-8
1-methyl-3-propyltrisulfane	17619-36-2
1-methyl-4-(prop-1-en-2-yl)benzene (*p*-cymenene)	1195-32-0

Table S1: Investigated key food odorants according to Dunkel et al. (2014)[1] (continued).

key food odorant	CAS#
1-octen-3-one	4312-99-6
2-(4-methylcyclohex-3-en-1-yl)propan-2-ol ((+/−)-α-terpineol), sum of isomers	98-55-5
2-(butan-2-yl)-3-methoxypyrazine	24168-70-5
2,3,5-trimethylpyrazine	14667-55-1
2,3-diethyl-5-methylpyrazine	18138-04-0
2,4-dimethylphenol	105-67-9
2,6-dimethoxyphenol (syringol)	91-10-1
2,6-dimethylphenol	576-26-1
2-[(1*R*/1*S*)-4-methylcyclohex-3-en-1-yl]propane-2-thiol ((*R*/*S*)-1-*p*-menthene-8-thiol), sum of isomers	71159-90-5
2-acetyl-2-thiazoline	29926-41-8
2-amino-1-phenylethan-1-one	613-89-8
2-ethyl-3,5(6)-dimethylpyrazine, sum of isomers	27043-05-6
2-ethyl-4-hydroxy-5-methyl-furan-3(2*H*)-one (homofuraneol)	27538-10-9
2*H*-1-benzopyran-2-one (coumarin)	91-64-5
2-methoxy-3-(propan-2-yl)pyrazine	25773-40-4
2-methoxy-4-(prop-2-en-1-yl)phenol (eugenol)	97-53-0
2-methoxy-4-[(*E*)-prop-1-en-1-yl]phenol (*trans*-isoeugenol)	97-54-1
2-methoxy-4-methylphenol (creosol)	93-51-6
2-methoxy-4-propylphenol	2785-87-7
2-methoxy-4-propylphenol	2785-87-7
2-methoxy-5-methylphenol	1195-09-1
2-methoxyphenol (guaiacol)	90-05-1
2-methyl-3-(methyldisulfanyl)furan	65505-17-1
2-methylbutanal	96-17-3

Table S1: Investigated key food odorants according to Dunkel et al. (2014)[1] (continued).

key food odorant	CAS#
2-methylbutanoic acid	116-53-0
2-methylfuran-3-thiol	28588-74-1
2-methylphenol (*o*-cresol)	95-48-7
2-methylpropanal (isobutyraldehyde)	78-84-2
2-methylpropanoic acid (isobutyric acid)	79-31-2
2-pentylpyridine	2294-76-0
2-phenylethan-1-ol	60-12-8
2-phenylethanethiol	4410-99-5
2-phenylethyl acetate	103-45-7
3-(methylsulfanyl)prop-1-ene	10152-76-8
3-(methylsulfanyl)propanal (methional)	3268-49-3
3-(methylsulfanyl)propan-1-ol (methionol)	505-10-2
(2*E*)-3-[(2*R*,3*R*)/(2*S*,3*S*)-3-pentyloxiran-2-yl]prop-2-enal (*trans*-4,5-epoxy-dec-2-enal)	13445431-2
3-[(prop-2-en-1-yl)disulfanyl]prop-1-ene (diallyl disulfide)	539-86-6
3-ethoxy-4-hydroxybenzaldehyde (ethylvanillin)	121-32-4
3-ethylphenol	620-17-7
3-hydroxy-2-methyl-4*H*-pyran-4-one (maltol)	118-71-8
3-hydroxy-4,5-dimethyl-furan-2(5*H*)-one (sotolon)	28664-35-9
3-methoxy-4-hydroxybenzaldehyde (vanillin)	121-33-5
3-methyl-1*H*-indole (skatol)	83-34-1
3-methylbutan-1-ol	123-51-3
3-methylbutanal	590-86-3
3-methylbutanoic acid	503-74-2
3-methylbutyl acetate	123-92-2

Table S1: Investigated key food odorants according to Dunkel et al. (2014)[1] (continued).

key food odorant	CAS#
3-methylheptan-4-one	15726-15-5
3-methylphenol	108-39-4
3-phenyl propanic acid	501-52-0
3-sulfanylhexan-1-ol	51755-83-0
3-sulfanylhexyl acetate	136954-20-6
4-(propan-2-yl)benzaldehyde (cuminaldehyde)	122-03-2
4-ethenylphenol	2628-17-3
4-ethyloctanoic acid	16493-80-4
4-ethylphenol	123-07-9
4-hydroxy-2,5-dimethylfuran-3(2*H*)-one (Furaneol®)	3658-77-3
4-methoxy-2-methylbutane-2-thiol	94087-83-9
4-methoxybenzaldehyde (*p*-anisaldehyde)	123-11-5
4-methyl-4-sulfanylpentan-2-one	19872-527
4-methylphenol	106-44-5
7-methoxy-2*H*-1-benzopyran-2-one (7-methoxycoumarin)	531-59-9
7-methyl-3-methylideneocta-1,6-diene (myrcene)	123-35-3
acetaldehyde	75-07-0
acetic acid	64-19-7
bis(methylsulfanyl)methane	1618-26-4
butan-1-ol	71-36-3
butane-2,3-dione	431-03-8
butanoic acid	107-92-6
butyl acetate	123-86-4
decanal	112-31-2
decanoic acid	334-48-5

Table S1: Investigated key food odorants according to Dunkel et al. (2014)[1] (continued).

key food odorant	CAS#
dimethyl trisulfane	3658-80-8
dipropyl trisulfide	6028-61-1
ethanol	64-17-5
ethyl (2*E*)-3-phenylprop-2-enoate (ethyl cinnamate)	103-36-6
ethyl (*3R*)-3-hydroxyhexanoate	2305-25-1
ethyl 2-methylbutanoate	7452-79-1
ethyl 2-methylpropanoate	97-62-1
ethyl 3-(methylsulfanyl)propanoate	13327-56-5
ethyl 3-methylbutanoate	108-64-5
ethyl 4-methylpentanoate	25415-67-2
ethyl acetate	141-78-6
ethyl benzoate	93-89-0
ethyl butanoate	105-54-4
ethyl cyclohexanecarboxylate	3289-28-9
ethyl decanoate	110-38-3
ethyl hexanoate	123-66-0
ethyl octanoate	106-32-1
ethyl pentanoate	539-82-2
ethyl phenylacetate	101-97-3
furan-2-ylmethanethiol (2-furfurylthiol)	98-02-2
heptan-2-ol	543-49-7
heptanal	111-71-7
hexan-1-ol	111-27-3
hexanal	66-25-1
hexanoic acid	142-62-1

Table S1: Investigated key food odorants according to Dunkel et al. (2014)[1] (continued).

key food odorant	CAS#
hexyl acetate	142-92-7
methyl (2*E*)-3-phenylprop-2-enoate (methyl cinnamate)	103-26-4
methyl (2*R*)-2/(2*S*)-2/3-methylbutanoate, sum of isomers	868-57-5
methyl 2-methylpropanoate	547-63-7
methyl benzoate	93-58-3
methyl butanoate	623-42-7
nonan-2-one	821-55-6
nonanal	124-19-6
octanal	124-13-0
octanoic acid	124-07-2
pent-1-en-3-one	1629-58-9
pentan-2,3-dione	600-14-6
pentanoic acid	109-52-4
phenylacetaldehyde	122-78-1
phenylacetic acid	103-82-2
propanal	123-38-6
propanoic acid	79-09-4
thiophen-2-ylmethanethiol	6258-63-5
undecanal	112-44-7

Table S2. Additionally investigated aldehydes.

aldehyde	CAS#
butanal	123-72-8
pentanal	110-62-3
dodecanal	112-54-9
tridecanal	10486-19-8
(2*E*)-but-2-enal	123-73-9
(2*E*)-pent-2-enal	1576-87-0
(2*E*)-oct-2-enal	2548-87-0
(6*Z*)-non-6-enal	2277-19-2
(4*E*)-dec-4-enal	65405-70-1
(2*E*)-dodec-2-enal	20407-84-5
(2*E*)-tridec-2-enal	7774-82-5
(2*E*,4*E*)-hexa-2,4-dienal	80466-34-8
(2*E*,4*E*)-hepta-2,4-dienal	4313-03-5
(2*E*,4*E*)-octa-2,4-dienal	5577-44-6
(2*E*,6*E*)-nona-2,6-dienal	17587-33-6
(2*E*,4*E*)-undeca-2,4-dienal	30361-29-6
(2*E*,4*E*)-dodeca-2,4-dienal	21662-16-8

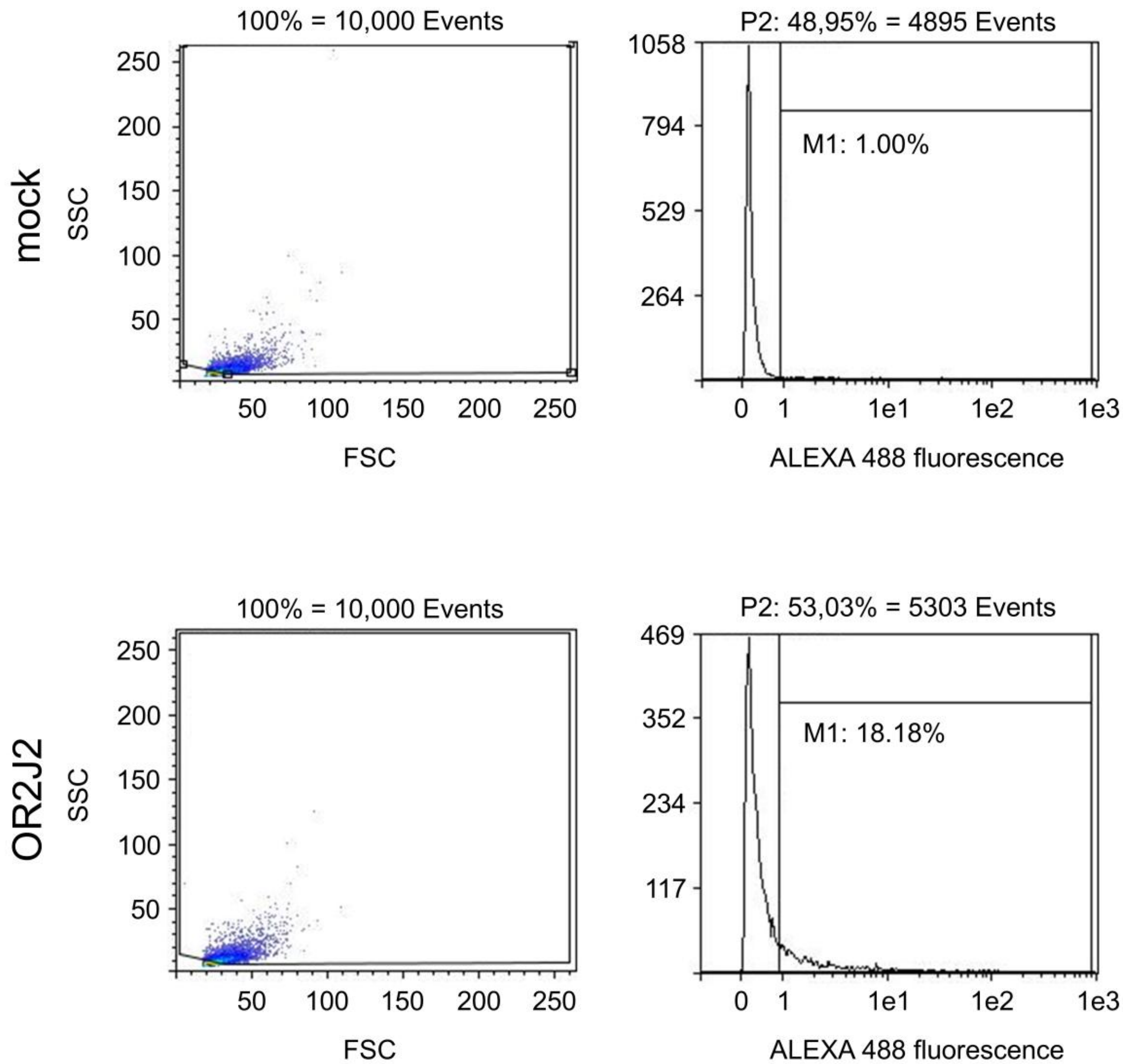

Figure S1. Gating strategy for cell surface expression analyses.

Flow cytometry data were analyzed using FlowLogic™ software (version 7.3, Inivai™, Mentone Victoria, Australia). A debris-exclusion gate (P2) was set on mock-transfected cells, and a positivity threshold (M2) was defined at ~1% of the gated population. These gates were then applied identically to all receptor-transfected cells. SSC, side scatter; FSC, forward scatter; ALEXA 488 fluorescence was detected via the FITC channel (excitation: 488 nm, emission: 525/550 nm) of the MACSQuant 16 flow cytometer (Miltenyi, Bergisch Gladbach, Germany).

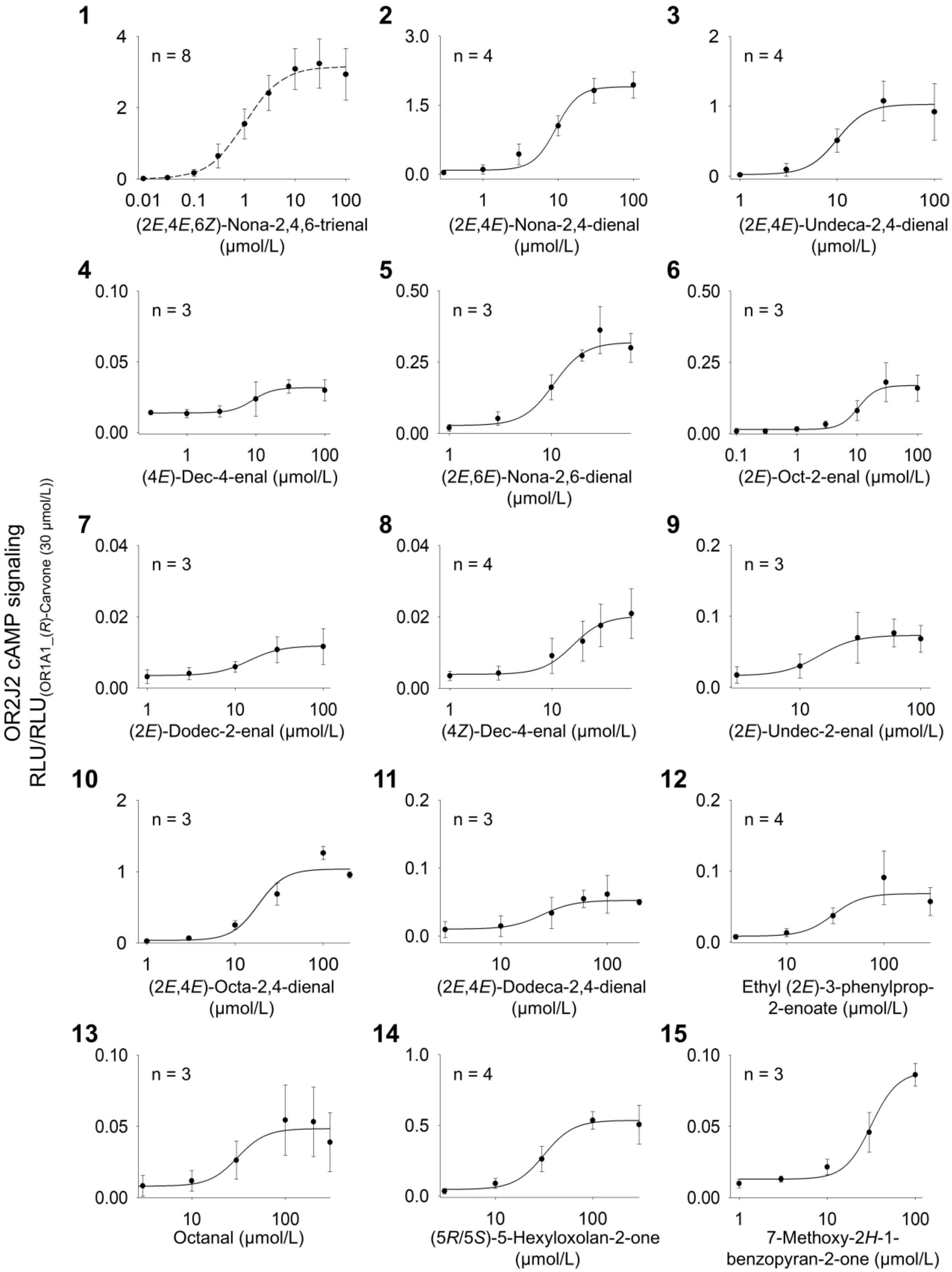

Figure S2. Concentration-response-relations of aldehyde and/or KFO agonists on OR2J2.

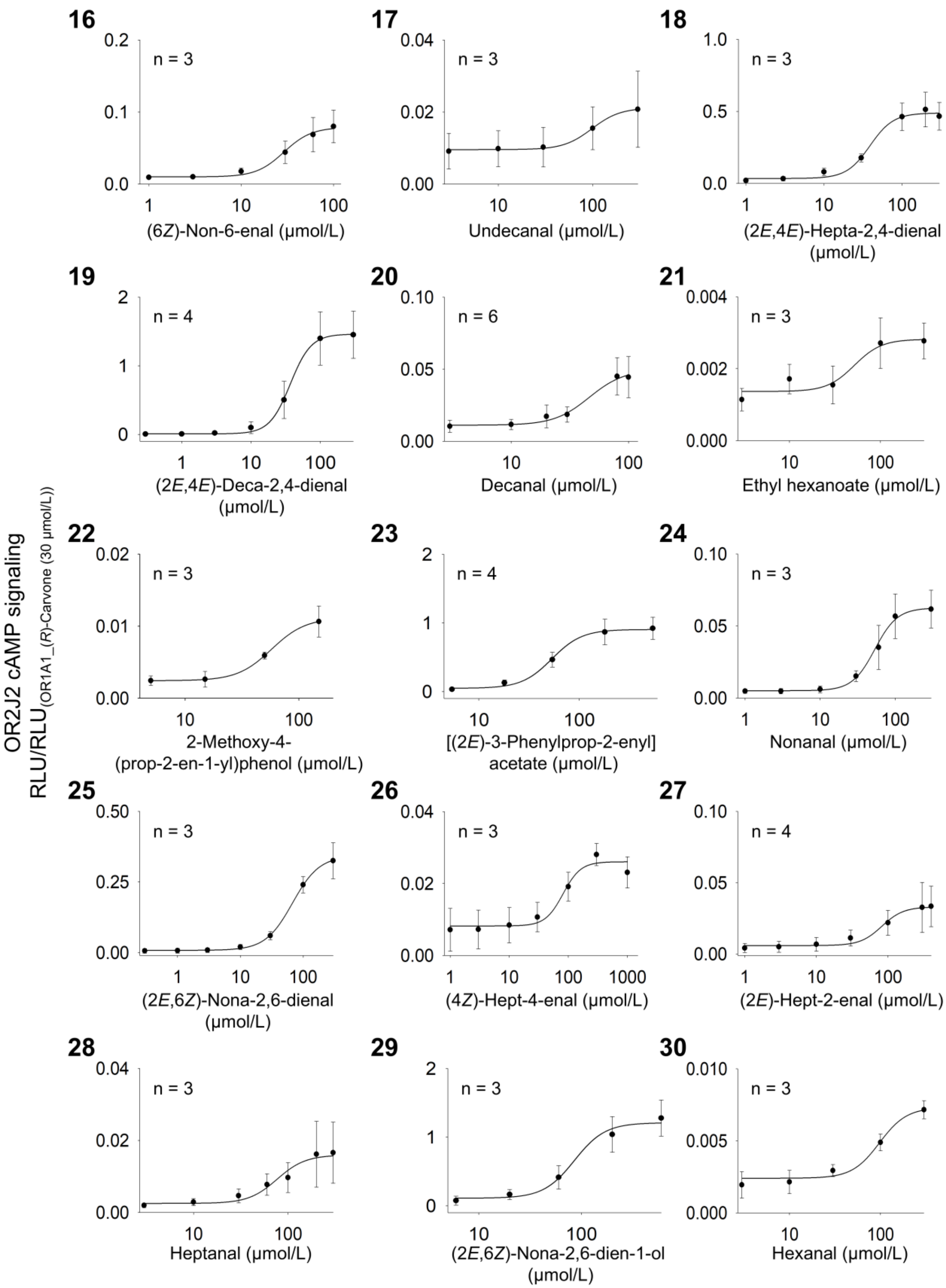

Figure S2. Concentration-response-relations of aldehyde and/or KFO agonists on OR2J2 (continued).

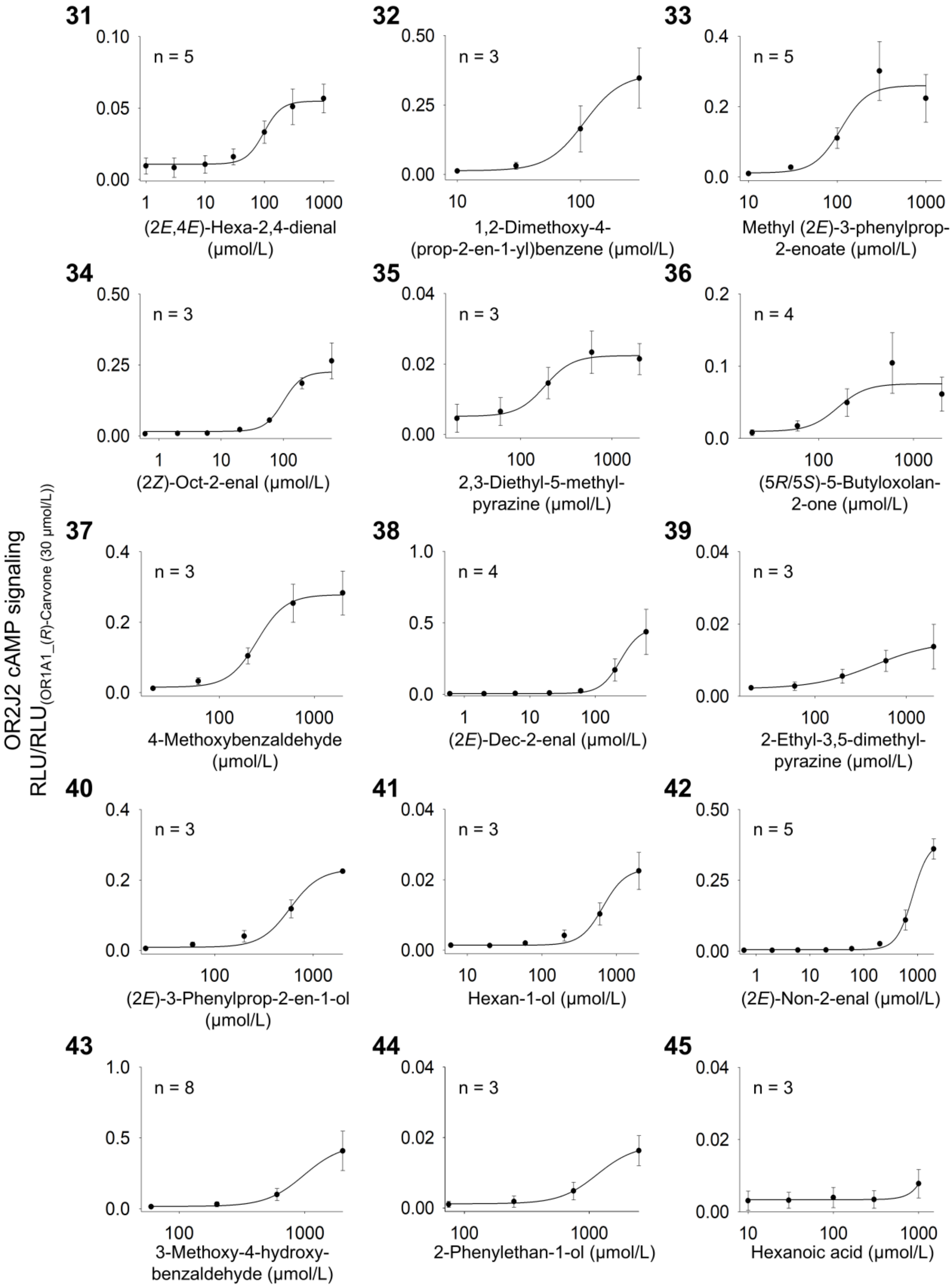

Figure S2. Concentration-response-relations of aldehyde and/or KFO agonists on OR2J2 (continued).

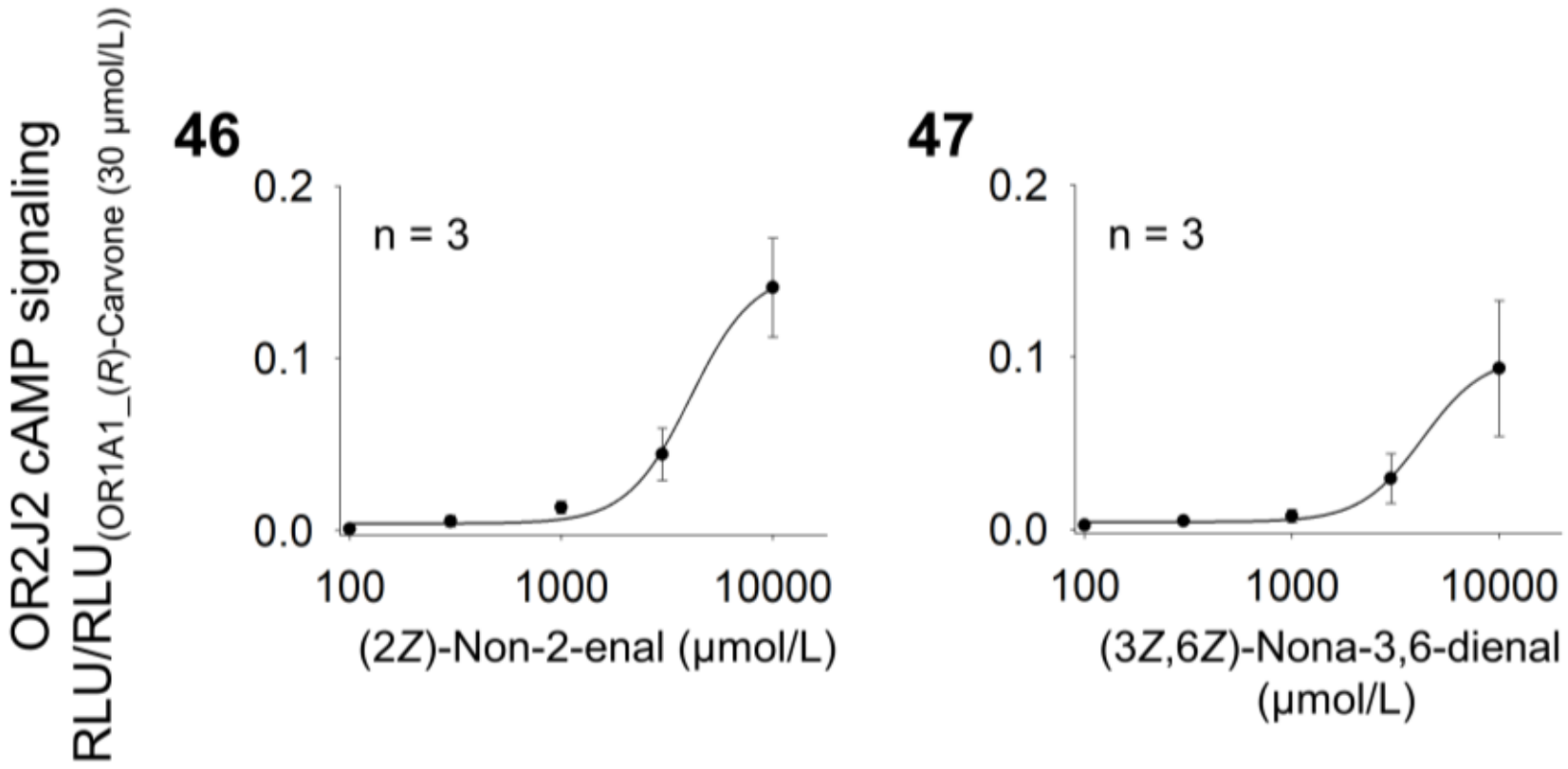

Figure S2. Concentration-response-relations of aldehyde and/or KFO agonists on OR2J2 (continued).

Concentration-response-relations of OR2J2 to (2*E*,4*E*,6*Z*)-nona-2,4,6-trienal (1), (2*E*,4*E*)-nona-2,4-dienal (2), (2*E*,4*E*)-undeca-2,4-dienal (3), (4*E*)-dec-4-enal (4), (2*E*,6*E*)-nona-2,6-dienal (5), (2*E*)-oct-2-enal (6), (2*E*)-dodec-2-enal (7), (4*Z*)-dec-4-enal (8), (2*E*)-undec-2-enal (9), (2*E*,4*E*)-octa-2,4-dienal (10), (2*E*,4*E*)-dodeca-2,4-dienal (11), ethyl (2*E*)-3-phenylprop-2-enoate (ethyl cinnamate;12), octanal (13), (5*R*/5*S*)-5-hexyloxolan-2-one ((4*R*/4*S*)-γ-undecalactone; 14), 7-methoxy-2*H*-1-benzopyran-2-one (7-methoxycoumarin; 15), (6*Z*)-non-6-enal (16), undecanal (17), (2*E*,4*E*)-hepta-2,4-dienal (18), (2*E*,4*E*)-deca-2,4-dienal (19), decanal (20), ethyl hexanoate (21), 2-methoxy-4-(prop-2-en-1-yl)phenol (eugenol; 22), [(2*E*)-3-phenylprop-2-enyl] acetate (cinnamyl acetate; 23), nonanal (24), (2*E*,6*Z*)-nona-2,6-dienal (25), (4*Z*)-hept-4-enal (26), (2*E*)-hept-2-enal (27), heptanal (28), (2*E*,6*Z*)-nona-2,6-dien-1-ol (29), hexanal (30), (2*E*,4*E*)-hexa-2,4-dienal (31), 1,2-dimethoxy-4-(prop-2-enyl)benzene (methyl eugenol; 32), methyl (2*E*)-3-phenylprop-2-enoate (methyl cinnamate; 33), (2*Z*)-oct-2-enal (34), 2,3-diethyl-5-methylpyrazine (35), (5*R*/5*S*)-5-butyloxolan-2-one ((4*R*/4*S*)-γ-nonalactone; 36), 4-methoxybenzaldehyde (*p*-anisaldehyde; 37), (2*E*)-dec-2-enal (38), 2-ethyl-3,5-dimethylpyrazine (39), (2*E*)-3-phenylprop-2-en-1-ol (cinnamyl alcohol; 40), hexan-1-ol (41), (2*E*)-non-2-enal (42), 4-hydroxy-3-methoxybenzaldehyde (vanillin; 43), 2-phenylethan-1-ol (44), hexanoic acid (45), (2*Z*)-non-2-enal (46), (3*Z*,6*Z*)-nona-2,6-dienal (47). Data were mock control-subtracted, normalized to the response of OR1A1 to (*R*)-carvone (30 µmol/L), and displayed as mean ± SD (n = 3–8). Numbers indicate the order of potency. RLU, relative luminescence units.

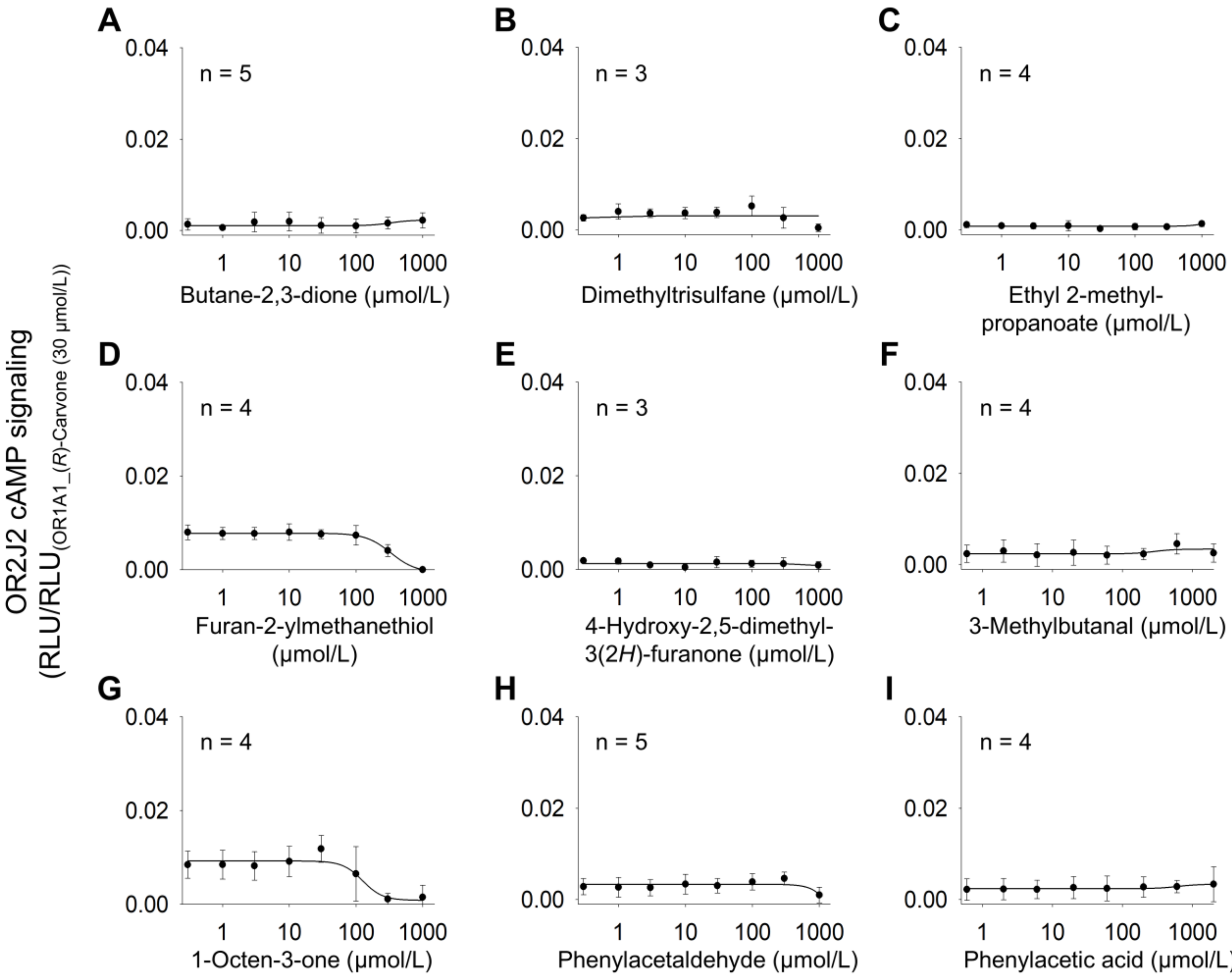

Figure S3. Validation of false-positive KFO hits on OR2J2.

Concentration-response-relations of OR2J2 to butane-2,3-dione (A), dimethyltrisulfane (B), ethyl 2-methyl-propanoate (C), furan-2-ylmethanethiol (D), 4-hydroxy-2,5-dimethylfuran-3(2*H*)-one (E), 3-methylbutanal (F), 1-octen-3-one (G), phenylacetaldehyde (H), phenylacetic acid (I). Data were mock control-subtracted, normalized to the response of OR1A1 to (*R*)-carvone (30 µmol/L), and displayed as mean ± SD (n = 3–5). RLU, relative luminescence units.

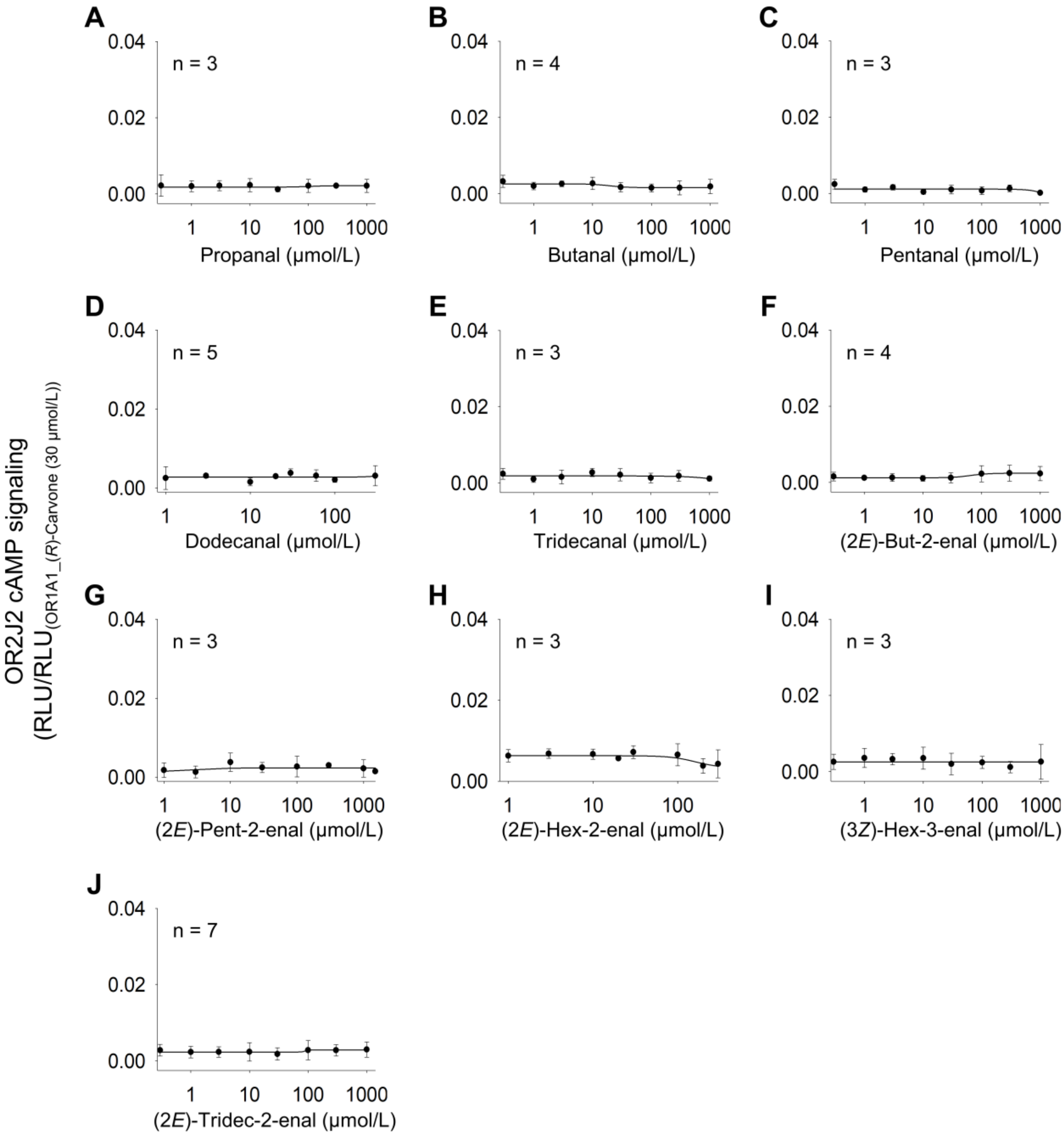

Figure S4. Certain aldehydes failed to activate OR2J2.

Concentration-response-relations of OR2J2 to propanal (A), butanal (B), pentanal (C), dodecanal (D), tridecanal (E), (2*E*)-but-2-enal (F), (2*E*)-pent-2-enal (G), (2*E*)-hex-2-enal (H), (3*Z*)-hex-3-enal (I), (2*E*)-tridec-2-enal (J). Data were mock control-subtracted, normalized to the response of OR1A1 to (*R*)-carvone (30 µmol/L), and displayed as mean ± SD (n = 3–7). RLU, relative luminescence units.

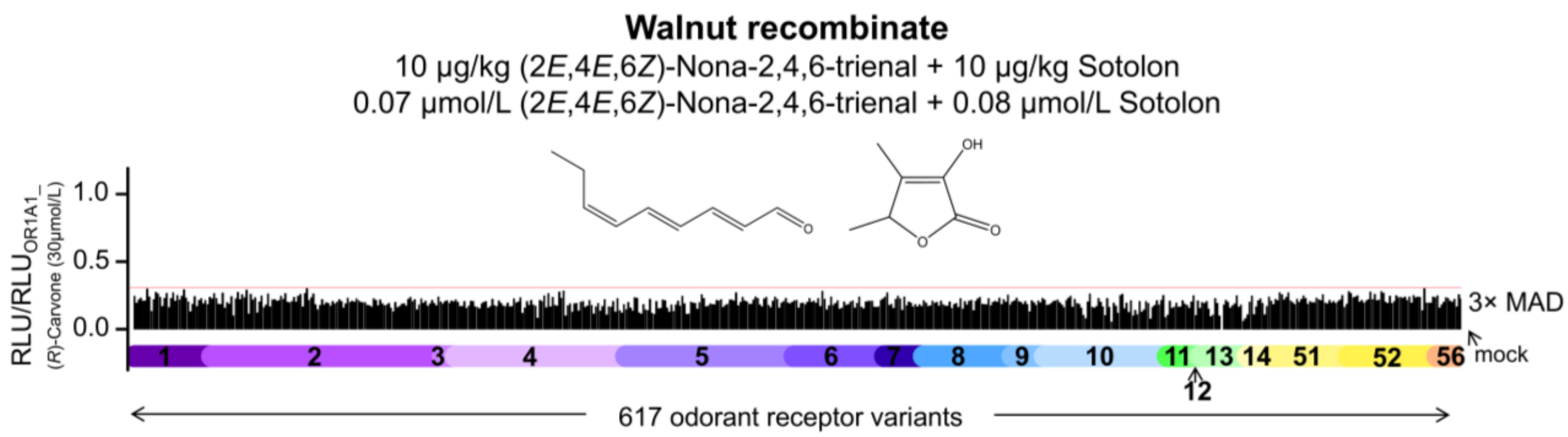

Figure S5. No receptor activation measurable at food grade concentrations.

Screening of 616 human OR variants with each 10 µg/kg (2*E*,4*E*,6*Z*)-nona-2,4,6-trienal and sotolon (1:1). Shown is the mean (n = 1 in duplicate). Data were normalized to the response of OR1A1 to (*R*)-carvone (30 µmol/L). The different OR families are color-coded and sorted in ascending order. The red line indicates the 3× MAD-threshold. RLU, relative luminescence units.

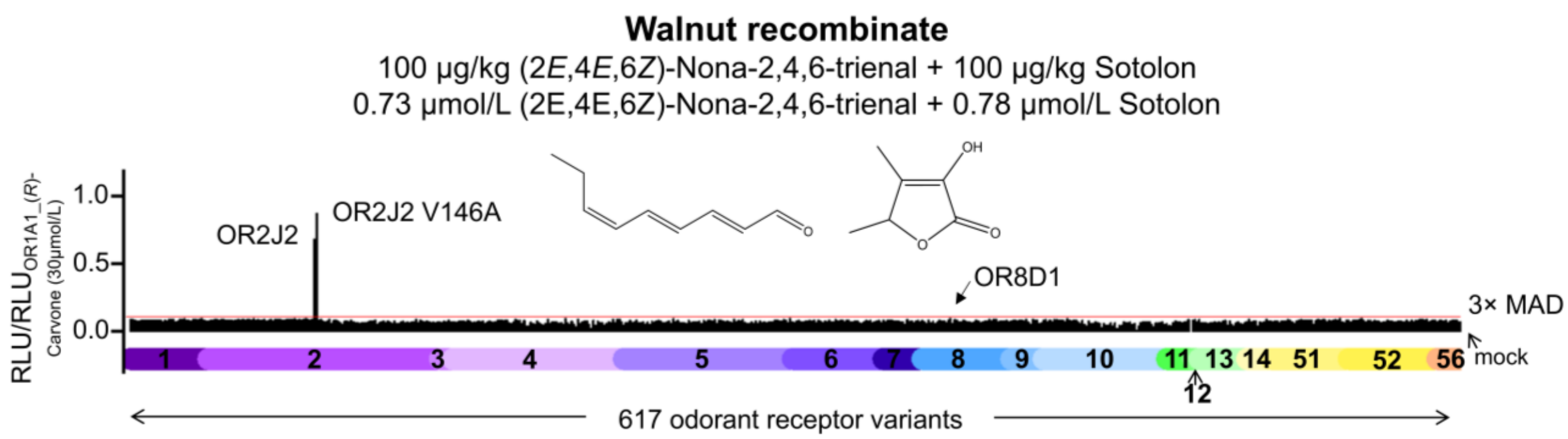

Figure S6. Activation pattern of walnut recombinate at 10× food grade concentrations.

Screening of 616 human OR variants with each 100 µg/kg (2*E*,4*E*,6*Z*)-nona-2,4,6-trienal and sotolon (1:1). Shown is the mean (n = 1 in duplicates). Data were normalized to the response of OR1A1 to (*R*)-carvone (30 µmol/L). The different OR families are color-coded. The red line indicates the 3× MAD-threshold. RLU, relative luminescence units.

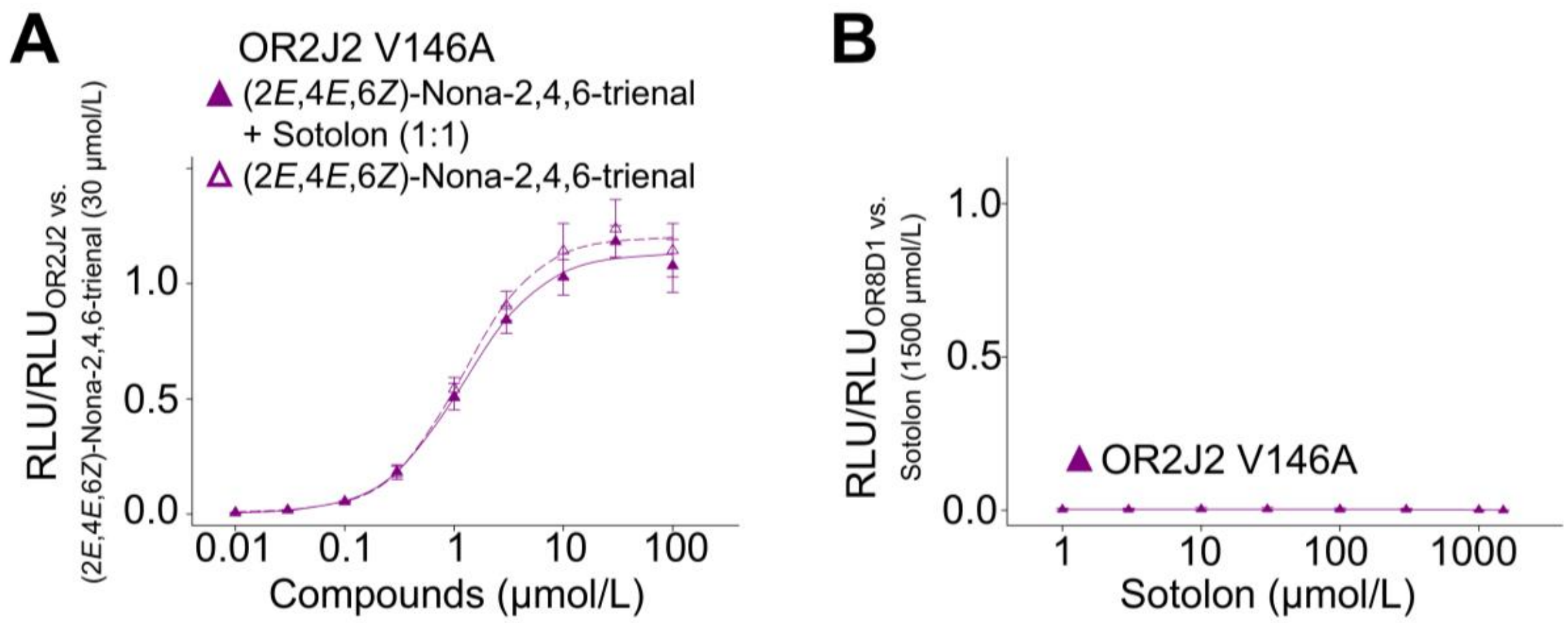

Figure S7. OR2J2 haplotype V146A is not activated or modulated by sotolon.

(A) Concentration–response relations of (2*E*,4*E*,6*Z*)-nona-2,4,6-trienal (open triangles) and the 1:1 mixture of (2*E*,4*E*,6*Z*)-nona-2,4,6-trienal and sotolon (filled triangles) on OR2J2 V146A. Data were mock control-subtracted, normalized to the response of OR2J2 ref. to (2*E*,4*E*,6*Z*)-nona-2,4,6-trienal (30 µmol/L), and displayed as mean ± SD (n = 5). (B) Concentration–response relations of sotolon on OR2J2 V146A. Data were mock control-subtracted, normalized to the response of OR8D1 to sotolon (1500 µmol/L) and displayed as mean ± SD (n = 6). RLU, relative luminescence units.

References

(1) Dunkel, A.; Steinhaus, M.; Kotthoff, M.; Nowak, B.; Krautwurst, D.; Schieberle, P.; Hofmann, T. Nature's Chemical Signatures in Human Olfaction: A Foodborne Perspective for Future Biotechnology. *Angew. Chem., Int. Ed.* **2014**, *53* (28), 7124–7143.
